# Calibration verification for stochastic agent-based disease spread models

**DOI:** 10.1371/journal.pone.0315429

**Published:** 2024-12-10

**Authors:** Maya Horii, Aidan Gould, Zachary Yun, Jaideep Ray, Cosmin Safta, Tarek Zohdi

**Affiliations:** 1 Mechanical Engineering Department, University of California, Berkeley, Berkeley, California, United States of America; 2 Data Sciences and Computing Department, Sandia National Laboratories, Livermore, California, United States of America; Beihang University, CHINA

## Abstract

Accurate disease spread modeling is crucial for identifying the severity of outbreaks and planning effective mitigation efforts. To be reliable when applied to new outbreaks, model calibration techniques must be robust. However, current methods frequently forgo calibration verification (a stand-alone process evaluating the calibration procedure) and instead use overall model validation (a process comparing calibrated model results to data) to check calibration processes, which may conceal errors in calibration. In this work, we develop a stochastic agent-based disease spread model to act as a testing environment as we test two calibration methods using simulation-based calibration, which is a synthetic data calibration verification method. The first calibration method is a Bayesian inference approach using an empirically-constructed likelihood and Markov chain Monte Carlo (MCMC) sampling, while the second method is a likelihood-free approach using approximate Bayesian computation (ABC). Simulation-based calibration suggests that there are challenges with the empirical likelihood calculation used in the first calibration method in this context. These issues are alleviated in the ABC approach. Despite these challenges, we note that the first calibration method performs well in a synthetic data model validation test similar to those common in disease spread modeling literature. We conclude that stand-alone calibration verification using synthetic data may benefit epidemiological researchers in identifying model calibration challenges that may be difficult to identify with other commonly used model validation techniques.

## Introduction

Accurate predictive modeling of disease spread is critical for understanding the potential impacts of an outbreak and implementing effective interventions. For example, models such as Covasim [1], OpenABM-Covid19 [2], CityCovid [3], and many others [4] have been used to inform policy decisions and intervention strategies in the recent COVID-19 pandemic. Considering the significant impact that these models can have on policy and on public perception of risk, it is important that they provide realistic predictions and uncertainty estimations.

Inaccuracies in model results may come from inherent randomness, simplifications and assumptions in model structure, or from errors in calibration of parameters. In this paper, we focus on the latter by testing parameter calibration methods on synthetic data generated by the same stochastic model we wish to calibrate. Synthetic data allows us to directly evaluate our parameter inference against known generative parameter values, a process we refer to as calibration verification.

We argue that a purely synthetic approach to calibration verification is valuable before tackling validation against real-world data. We will refer to processes in which model results are compared to real-world data for validation purposes as “overall model validation”. In overall model validation, several factors can be out of the modeler’s control to some degree—the quality and amount of data, the prior assumptions, the model form error, etc. Some overall model validation techniques may be unable to identify the specific source of errors since calibration, model structure, prior choice, and real-world data all feed into results. Additionally, common overall model validation methods like posterior predictive checks are often intended primarily for model criticism and not calibration verification in particular, and may have low power in disease spread applications (in other words, limited ability to identify incorrect models) [5]. Synthetic data testing for calibration verification isolates a controllable section of the process—the estimation of parameters through calibration—and helps safeguard against preventable errors that may otherwise be difficult to identify with common overall model validation techniques. In doing so, it also reduces uncertainty when moving on to model validation, as confidence in calibration results allows model validation to focus on other sources of error.

Previous work in the disease spread modeling field has typically lumped calibration verification into overall model validation. In this paper, we will demonstrate that stand-alone calibration verification tests can serve as a powerful tool for disease spread modelers, enabling researchers to more easily identify errors and their sources. In particular, we will investigate simulation-based calibration (a particular type of calibration verification) through tests on two different calibration methods and comparisons to posterior predictive checks (a common type of overall model validation).

This paper is organized as follows. The Literature review section provides an overview of the literature relevant for our work. The Methods section contains a description of the agent-based model (in the section titled “Agent-based model framework”) and the Bayesian inference framework (in the section titled “Bayesian inference”). The model calibration results are presented in the Results section, followed by the Discussion and Conclusion. Additional information is presented in the Supporting Information.

## Literature review

We use an agent-based model (ABM), which is a commonly-used epidemiological model structure where individuals in a population are represented as unique agents, each with a set of characteristics and behavioral rules [6]. Compartmental ABMs categorize agents into bins for disease status tracking—for example, we use compartments of susceptible (S), exposed (E), infected (I), and removed/recovered (R), which together form an SEIR model. ABMs are generally stochastic due to randomness in agent behavior, position, and/or infection mechanisms, and are well-suited to capturing the effects of heterogeneous population spread. This is in contrast to compartment-based ordinary differential equation (ODE) models, which are a widely-used model type, and are generally deterministic. The ODE approach typically assumes homogeneous mixing within a population, which can limit complexity and realism [7]. Given their widespread popularity, there is much recent work exploring the use of ABMs and applicable calibration methodologies, including techniques like surrogate modeling with ODEs, IPDEs (integro partial differential equations), and SDEs (stochastic differential equations) [8, 9]; differentiable ABMs [10]; and neural-network based posterior inference [11].

### Calibration verification in epidemiological modeling

In the literature focused on disease spread modeling, verification of calibration methods is typically lumped into the overall model validation. Commonly, a confidence or credible interval on simulation results will be plotted along with the observed data to indicate some agreement between the model and reality. The intervals may be extracted from posterior predictive distributions (resulting in a visual posterior predictive check) in cases where calibration is performed via Bayesian inference [3, 12–16], or otherwise based on repeated stochastic simulations at a fixed best-fit parameter or parameter set [1, 2, 17, 18]. The former accounts for both stochastic characteristics in the model and uncertainty of the parameters, while the latter only reflects stochastic behavior of the model. This visual evaluation of fit is sometimes supplemented with a quantification of discrepancies between data and model predictions, i.e., root mean squared error [13], marginal log-likelihoods [13], quantitative posterior predictive checks [19], etc.

**Fig 1:**
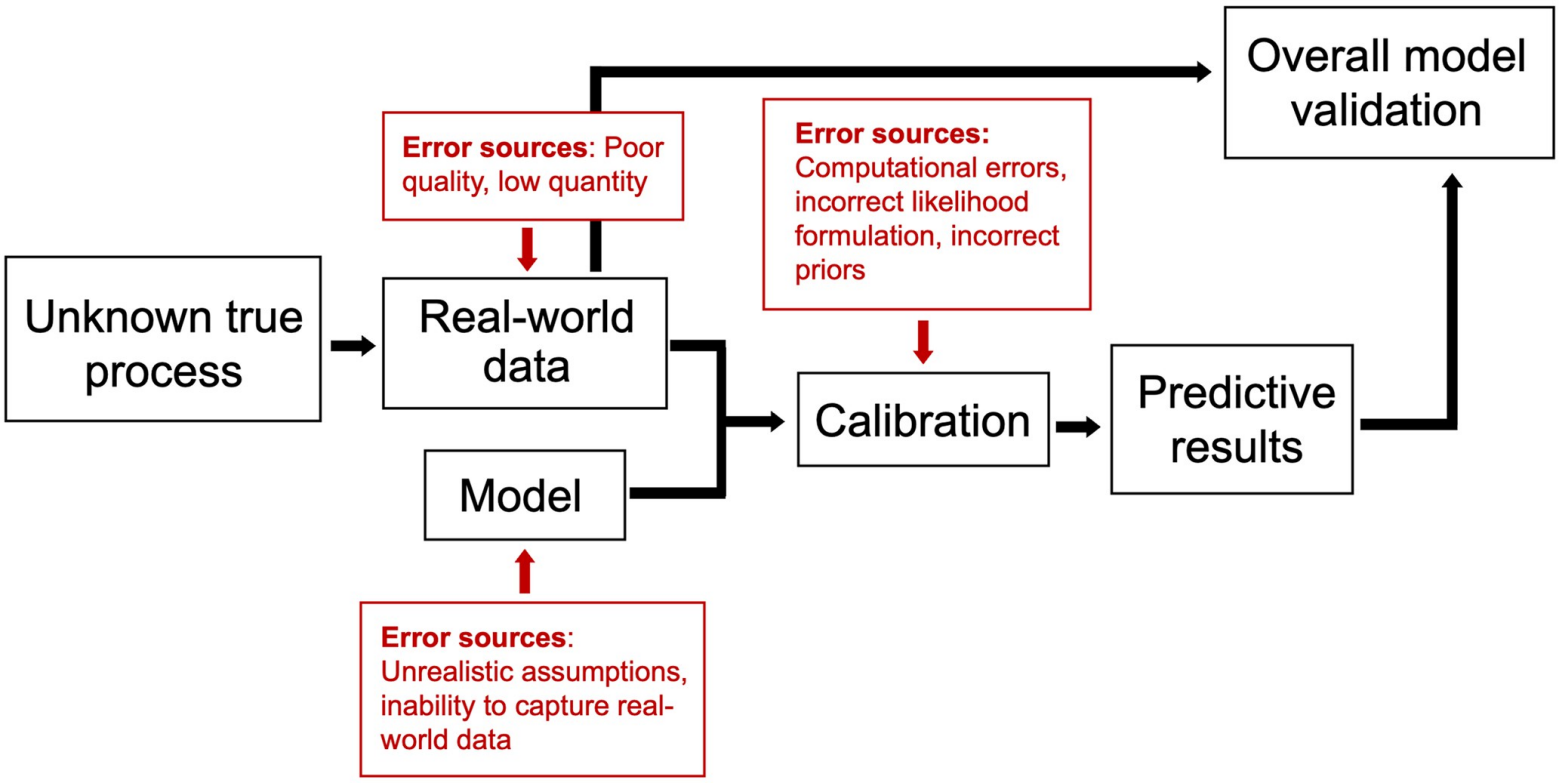
Diagram of overall model validation and associated error sources.

**Fig 2:**
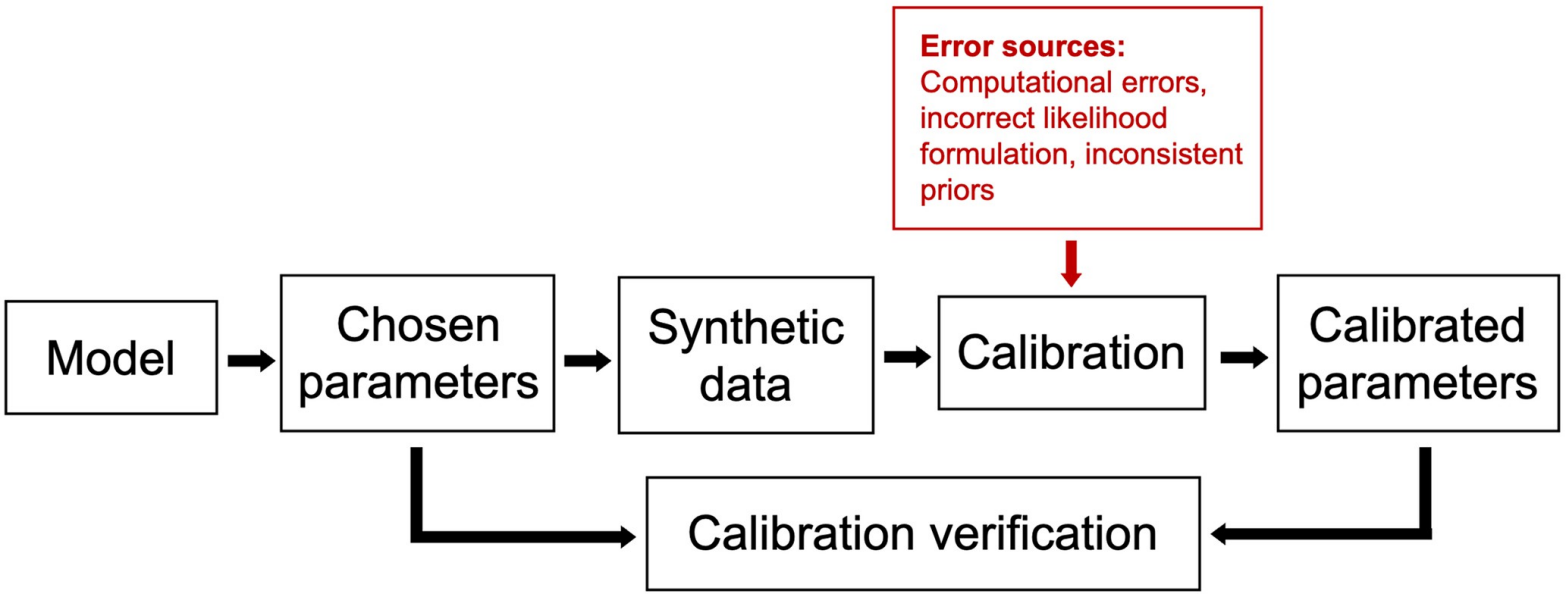
Diagram of calibration verification using synthetic data and associated error sources.

Since a posterior predictive check is the result of both calibration and model execution, it can identify errors stemming from a range of sources, including computational issues, incorrect priors, incorrect likelihoods (i.e., likelihoods inconsistent with the generative model), and incorrect models (i.e., generative models incapable of representing observed data, or otherwise inaccurate). While this broadness is valuable, it may also make it difficult to determine the primary cause of errors. Isolating the calibration verification process by testing calibration on synthetic data allows for more controlled investigation of error sources, as error associated with real-world data and/or model structure are eliminated. An illustration of the error sources in overall model validation and calibration verification are shown in Figs 1 and 2, respectively.

Additionally, in the absence of independent calibration verification, visual posterior predictive checks may disguise calibration issues. Data re-use in calibration and validation is known to lead to low power (in other words, limited ability to identify incorrect models) in posterior predictive checks [5]. Holding out a portion of the data so that the model is only calibrated with a portion of the data may improve power, but held-out data is still generally not independent from data used for calibration in epidemiological applications. Evaluation of visual posterior predictive checks are also usually qualitative, without a clear decision process to indicate the line between acceptable and unacceptable results.

Given these challenges, we explore the use of simulation-based calibration (SBC) to verify Bayesian inference algorithms using synthetic data.

### Simulation-based calibration

An intuitive starting place for synthetic data testing of calibration verification is to do some version of the following: 1.) choose parameter set values, 2.) generate synthetic data by running the chosen model with those parameters, 3.) attempt to guess the original parameters by running calibration on the synthetic data, and 4.) compare the guessed parameter estimates to the originally chosen parameters. Some measure of “agreement” should be found between the estimates and originally chosen parameters if the calibration process is sound—this is the basic idea behind simulation-based calibration.

The procedure for SBC is shown in Algorithm 1. First, parameter set values $\tilde{\theta }$ are drawn from the prior ($\tilde{\theta }∼p\left(\theta \right)$) and used to generate a model result, $\tilde{y}$ (where $\tilde{y}∼p\left(y|\tilde{\theta }\right)$, since the model is stochastic). Calibration is used to “guess the parameters” by predicting a posterior, $p(\theta |\tilde{y})$, given $\tilde{y}$. The posterior is then compared to the originally chosen parameters by drawing *L* posterior samples, {*θ*_1_, …, *θ*_*L*_} (as is naturally done in MCMC), and calculating the rank statistic of $\tilde{\theta }$ with respect to the posterior samples. Given that we have *L* draws from the posterior in each repetition of the process, ranks can take on integer values between 0 and L (0 ≤ *rank* ≤ *L*, giving L+1 possible values). If the Bayesian inference process has been implemented correctly, the ranks of $\tilde{\theta }$ with respect to {*θ*_1_, …, *θ*_*L*_} should be draws from the discrete uniform distribution along the (L+1) possible ranks.

**Algorithm 1** Simulation-based calibration

1: **for** i = 1 to n **do**

2:  Draw a sample from the prior: $\tilde{\theta }∼p\left(\theta \right)$

3:  Run the model using parameter $\tilde{\theta }:\tilde{y}∼p\left(y|\tilde{\theta }\right)$

4:  Draw samples from posterior: $\left\{\theta_{1},\ldots ,\theta_{L}\right\}∼p\left(\theta |\tilde{y}\right)$

5:  Determine rank of $\tilde{\theta }$ with respect to {*θ*_1_, …, *θ*_*L*_}: $rank(\left\{\theta_{1},\ldots ,\theta_{L}\right\},\tilde{\theta })$

6: **end for**

To justify this claim, observe that $\left(\tilde{y},\tilde{\theta }\right)$ represents a draw from the joint distribution *p*(*y*, *θ*) (since *p*(*y*, *θ*) = *p*(*y*|*θ*)*p*(*θ*)), and therefore $\tilde{\theta }$ represents a draw from the conditional posterior distribution $p(\theta |\tilde{y})$ [20].

The theoretical basis of simulation-based calibration is concerned with the property of inferences being “well-calibrated”—in this usage, being “well-calibrated” does not refer to an individual correct choice of parameters to fit given data, but to a particular type of statistical consistency between posterior inferences and the true posterior. An illustrative example, given in Ref. [21, 22], goes as follows: a weather forecaster who reports a predicted probability of rain, *ω*, is “well-calibrated” if the proportion of days with rain is *ω* for days where the forecaster predicted a probability of *ω*. This is described mathematically as “probabilistic calibration” in [23]. Simulation-based calibration is a way of testing if a model is probabilistically calibrated.

A version of this method was first developed by Cook, Gelman, and Rubin [20]. In that work, estimated quantile values are used instead of rank statistics, where $\hat{q}\left(\tilde{\theta }\right)=\frac{1}{L}\sum_{i=1}^{L}1\left(\tilde{\theta }>\theta_{i}\right)$. This is equivalent to calculating the empirical CDF value ${\hat{F}}_{\theta |\tilde{y}}\left(\tilde{\theta }\right)$. They suggest that a continuous uniform distribution of estimated quantile values is a necessary condition for a correctly implemented posterior inference process.

One issue with this method, as pointed out by Talts [24], is that the discrete nature of the quantile estimates can cause artifacts. In the extreme, imagine a test set-up with L = 1. Quantile estimates can only take on values of 0 or 1 in this case, which cannot result in a *continuous* uniform distribution. It can be shown that quantile values calculated using the *exact* CDF, $q\left(\tilde{\theta }\right)=F_{\theta |\tilde{y}}\left(\tilde{\theta }\right)=\int p\left(\theta |\tilde{y}\right)1\left(\tilde{\theta }>\theta \right)d\theta$, will be continuously uniformly distributed given continuous posteriors, but this does not hold for quantile *estimates* [20, 24]. Additionally, if samples {*θ*_1_, …, *θ*_*L*_} are collected using MCMC, a common method for evaluating Bayesian posteriors, autocorrelation is expected between samples. The proof of uniformity of quantile values relies on independence of samples, so this must be addressed before using the method with MCMC sampling. Talts [24] expands on the work of Cook, Gelman, and Rubin by switching from quantile estimates to rank statistics. Then, the ranks of $\tilde{\theta }$ with respect to {*θ*_1_, …, *θ*_*L*_} are expected to be discretely uniformly distributed across the integers [0, *L*] for correctly implemented inference processes. Thinning is used to remove autocorrelation for MCMC samples.

These methods resemble techniques used for ensemble forecast evaluation, such as probability integral transform (PIT) values and rank histograms. PIT values are calculated as the quantile of observed data with respect to a predictive forecast CDF, such that if the predictive forecast CDF is ideal (“ideal” defined as being the same as the true, underlying generative CDF), the PIT values will be uniformly distributed [23] (note that in comparison to the SBC procedure, where use of estimated quantiles can lead to artifacts, PIT values are calculated using an exact CDF, rather than an empirical CDF generated with finite samples). Similarly, rank histograms generally determine the rank of observed values with respect to ensemble predictions, with uniform ranks expected for reliable ensembles [25].

As discussed at length in previous literature, uniformity of a rank histogram of PIT values is a necessary but not sufficient condition for ideal forecasters [23]. The same is true for simulation-based calibration—given a correctly implemented posterior inference process, it follows that the distribution of ranks of $\tilde{\theta }$’s with respect to posterior samples ({*θ*_1_, …, *θ*_*L*_}) will be discretely uniform (*Unif*(0, *L*)). However, an incorrect posterior inference process can also result in a uniform distribution of ranks—for instance, if samples {*θ*_1_, …, *θ*_*L*_} were simply drawn from the prior, the ranks of $\tilde{\theta }$ with respect to {*θ*_1_, …, *θ*_*L*_} would also represent draws from a discrete uniform distribution (*Unif*(0, *L*)).

It is also important to note that SBC is a method to check an inference process for the *given Bayesian model*, i.e., the likelihood (implied by the chosen generative model) and prior [20, 24]. SBC can identify errors caused by computational issues in the posterior calculation, and errors caused by issues with the likelihood function definition (e.g., if the likelihood function used to calculate the posterior is inconsistent with the likelihood implied by the generative model). It can also identify a possible mismatch between the prior used in the posterior calculation and the one used in the SBC procedure (Algorithm 1, line 2), but it will not be able to guarantee robustness to choice of prior, which would require further sensitivity analysis. It is also not able to assess model correctness, or the ability of the model to generate data similar to real-world data, as it uses synthetic data (data generated by the model in question) for comparisons. These concerns require an overall model validation approach, which could include techniques like posterior predictive checks [26]. Due to these limitations, sensitivity checks and overall model validation are important parts of a full modeling procedure. While this paper focuses on synthetic data testing for calibration verification, many recent papers have explored the practical applications of model calibration to problems with experimental data [27–29].

In summary, overall model validation makes comparisons between model results and real-world data, which serves as a test of calibration framework, model fitness, choice of prior, and discrepancy between model prediction and data. On the other hand, SBC focuses only on testing calibration by making repeated comparisons between posterior distributions inferred from synthetic data and the parameter values used to generate that synthetic data. In disease spread modeling literature, overall model validation is typically used as a catch-all check without additional reported tests on calibration [1–3, 12–18]—however, isolating calibration verification with techniques like SBC could allow modelers to more easily pinpoint the source of both calibration and model issues. In this paper, we will demonstrate how SBC can be used to identify calibration issues that may otherwise be difficult to identify with an overall model validation approach, showing that SBC can serve as a powerful tool for disease spread modelers.

## Methods

**Fig 3:**
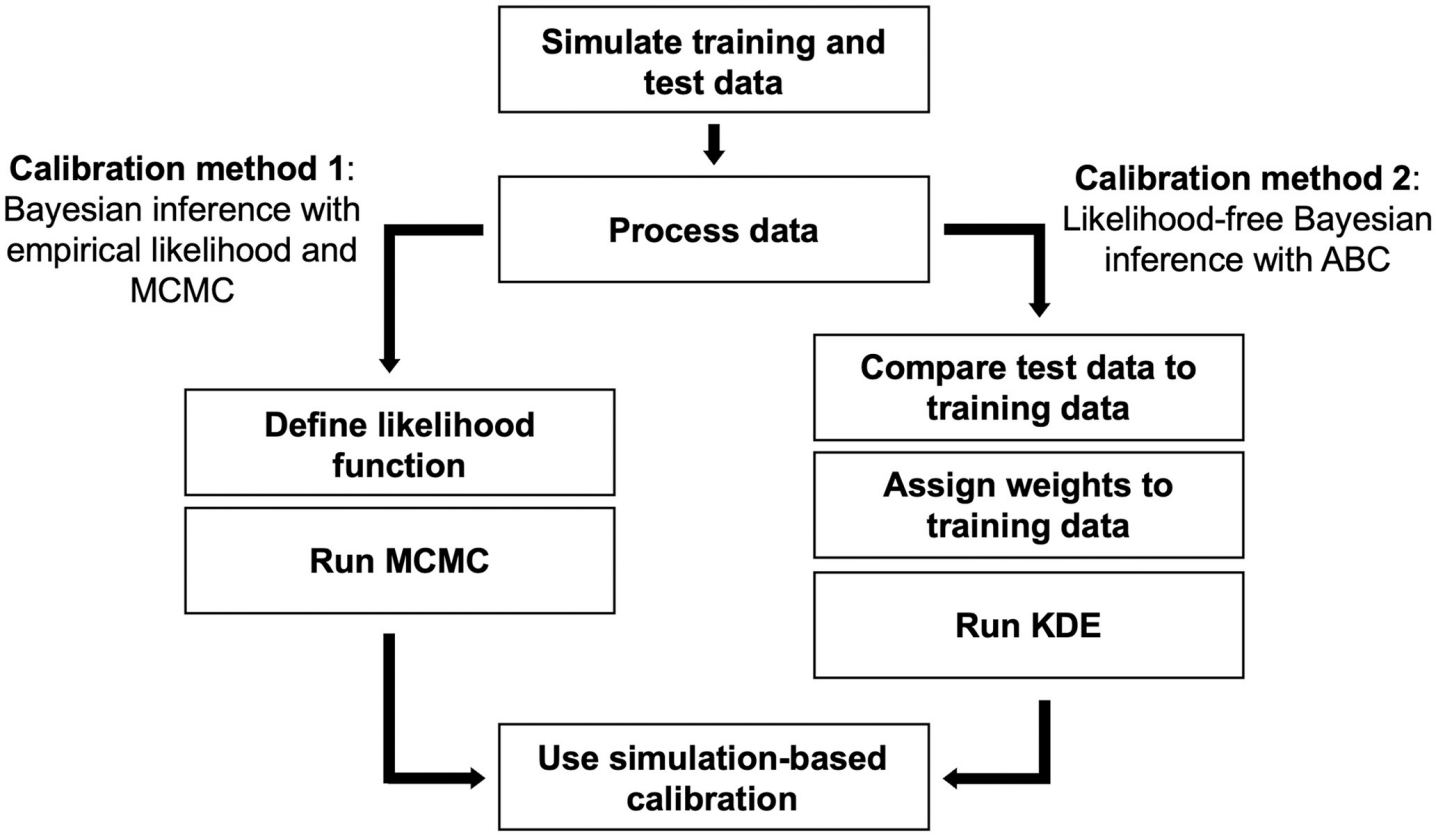
Flowchart of calibration verification process using synthetic data.

In this section, we describe our methodology, including the creation of our ABM, calibration methods, and verification with SBC. The overall process is shown in Fig 3. The process begins with generating training and test data using the ABM, which is then processed into summary statistics. We employ Bayesian inference to calibrate the model. In calibration method 1, the training data is used to define an empirical likelihood function, which is used with MCMC to generate posteriors. For calibration method 2, calibration on a given test data sample is performed by comparing the sample against all training data samples using a scoring process, which allows us to assign weights to each training data sample (with “similar” matches weighted highly, “dissimilar” matches weighted low or zero), and finally use kernel density estimation (KDE) to convert discrete scores into a continuous posterior. Finally, the results of calibration with both methods are evaluated according to SBC.

We will run calibration tests for two scenarios: the first will have only one parameter to calibrate, the second will have two, and will be referred to as the “one-parameter case” and “two-parameter case” respectively.

### Agent-based model framework

**Fig 4:**
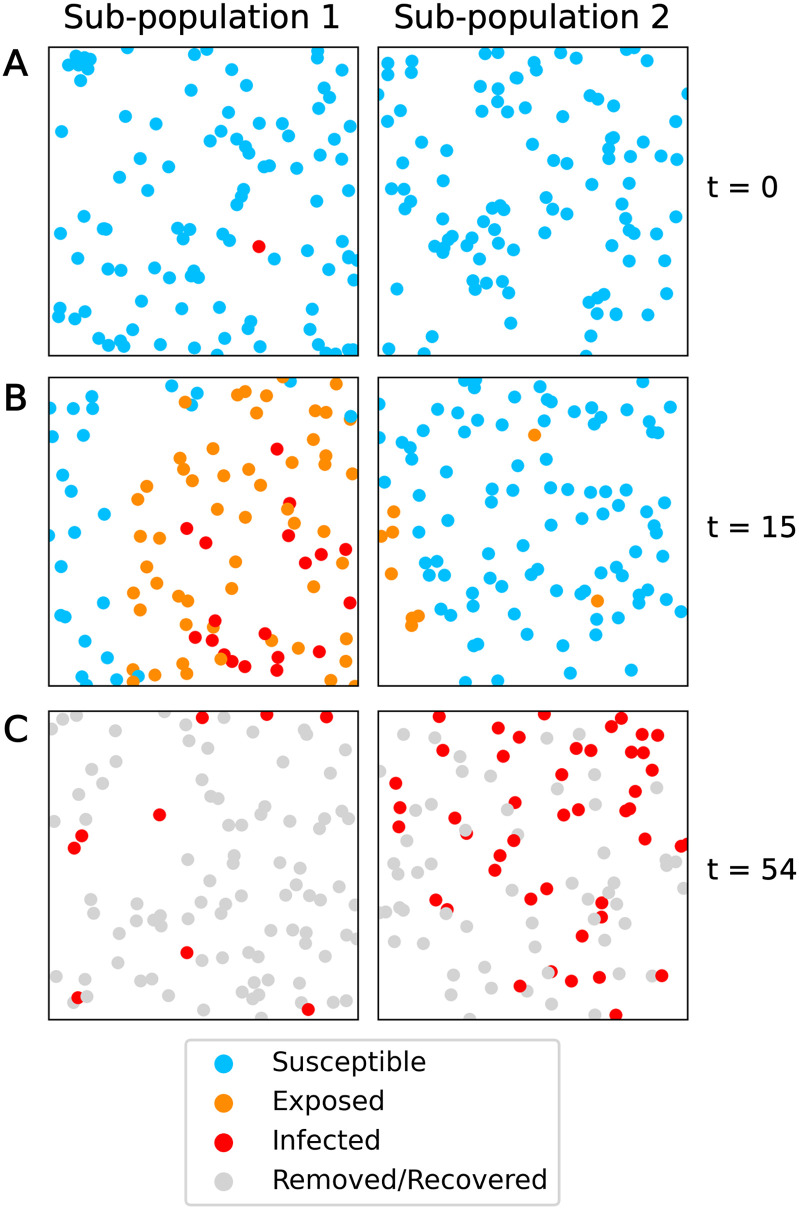
ABM example results. ABM results for evolution of disease spread in two sub-populations, beginning with one initially infected individual in sub-population 1. The radius of the dots is the infection distance. (A) t = 0. (B) t = 15. (C) t = 54.

Following a similar approach developed in Zohdi [30], we implement an agent-based modeling scheme (Fig 4). Agents move within a 2D space (denoted by the Cartesian *x*_1_ and *x*_2_ directions), and are confined within one or many rectangular domains representing sub-populations. Each domain is defined by limits *x*_1,*low*_, *x*_1,*high*_, *x*_2,*low*_, *x*_2,*high*_.

#### Agent motion

Agents move at each time step in a random direction with random velocity according to Eq 1,

$$\begin{array}{c} x_{i}\left(t+\Delta t\right)=x_{i}\left(t\right)+M*\Delta t*\delta_{x_{i}} \end{array}$$ (1)

where the agent’s position is ***r*** = (*x*_1_, *x*_2_), Δ*t* is the time step, *M* is the mobility parameter in units of distance per unit time, and $\delta_{x_{i}}$ is a random number sampled from a uniform distribution between −1 and 1 ($\delta_{x_{i}}∼U\left(-1,1\right)$). Higher mobility parameters allow agents to move more quickly, which accelerates the rate of disease spread. The agents move independent of the motion of the other agents, and agents are not allowed to move outside of their domains. After implementing the movement, if an agent is outside its domain, it is reflected back according to Algorithm 2.

**Algorithm 2** Agent bouncing algorithm

1: **for** i = 1 to 2 **do**

2:  **while** x[i] < x_low[i] ∨ x[i] > x_high[i] **do**

3:   **if** x<x_low **then**

4:    x[i] = 2*x_low[i]-x[i]

5:   **else**

6:    x[i] = 2*x_high[i]-x[i]

7:   **end if**

8:  **end while**

9: **end for**

When there are multiple sub-populations, agents may “jump” between domains based on a jumping probability parameter, *J*. At each time step, a random number between 0 and 1 is sampled (∼ *U*(0, 1)): if it is less than the jumping probability parameter *J*, the agent is moved to a random position within a random new sub-population. If it is higher, the agent remains in its current sub-population.

#### Agent states

Our framework tracks whether an agent is Susceptible (S), Exposed (E), Infected (I), or Removed/Recovered (R). When an infected agent *i* is within infection distance *D*_*I*_ of a susceptible agent *j*, that susceptible agent becomes exposed (‖***r***_*i*_ − ***r***_*j*_‖_2_ < *D*_*I*_, where ***r***_*i*_ and ***r***_*j*_ are the position vectors of agents *i* and *j*). An exposed agent becomes infected after exposure time *t*_*e*_, and an infected agent becomes recovered after infection time *t*_*i*_, at which point they no longer expose any nearby susceptible agents and are not able to be reinfected. The values of *t*_*e*_ and *t*_*i*_ are sampled from gamma distributions and are unique to each agent—they are assigned at the beginning of the simulation, and are fixed throughout. All agent states are tracked throughout the simulation. This data is summarized as the total number of S, E, I, R, and new infected agents per sub-population at each time step. We track status counts based on an agent’s original sub-population.

#### Simulation workflow

The general algorithm of our ABM simulation is described in Algorithm 3.

**Algorithm 3** Overall ABM algorithm

1: // **Initialize sub-population(s):**

2: Generate random initial positions for each agent

3: Assign initial disease states (S, E, I, or R) based on inputted initial fractions

4: Assign values of *t*_*e*_ and *t*_*i*_ to each agent

5: // **Step forward in time until simulation duration**, *t*_*Sim*_, **is reached**:

6: **for** t = 0 to *T*/Δ*t* **do**

7:  **if** using multiple sub-populations **then**

8:   Update agent positions via jumping between sub-populations

9:  **end if**

10:  Apply motion (Eq 1)

11:  Enforce domain boundaries (Algorithm 1)

12:  Update agent states based on time parameters *t*_*e*_ and *t*_*i*_

13:  Check distance between infected agents and susceptible agents

14:  **for** susceptible agents within infection radius **do**

15:   Update agent state to infected

16:  **end for**

17:  Save agent state data

18: **end for**

The simulation inputs are summarized in Table 1. Exposure time, *t*_*e*_, and infection time, *t*_*i*_, are not included in the table, as they are randomly sampled for each agent from gamma distributions according to respective mean and standard deviation values *μ*_*e*_, *σ*_*e*_, *μ*_*i*_, *σ*_*i*_. Two simulations are described: in the one-parameter case, the simulation has a single sub-population, while in the two-parameter case, the simulation has two sub-populations. In the former, the parameter of interest for calibration is mobility, while in the latter, we will calibrate both mobility and jumping probability. A simulation for the two-parameter case (200 agents) runs in less than a second on a laptop. The values are non-dimensional, having been normalized by their respective units of length, time, etc.

**Table 1:** Parameters used in ABM.

Symbol	Parameter	One-parameter case input values	Two-parameter case input values
Δ*t*	Time step	0.1	0.1
*T*	Total length of simulation	300	300
*m*	Total number of sub-populations	1	2
***x***_**1,*low***_	Lower x-direction domain boundaries	0	(0, 0.2)
***x***_**1,*high***_	High x-direction domain boundaries	0.1	(0.1, 0.3)
***x***_**2,*low***_	Lower y-direction domain boundaries	0	(0, 0)
***x***_**2,*high***_	High y-direction domain boundaries	0.1	(0.1, 0.1)
***N***_***pop***_	Sub-population sizes	100	(100, 100)
***S***_**0**_	Initial fractions of susceptible agents	0.99	(0.99, 1)
***E***_**0**_	Initial fractions of exposed agents	0	(0, 0)
***I***_**0**_	Initial fractions of infected agents	0.01	(0.01, 0)
***R***_**0**_	Initial fractions of recovered agents	0	(0, 0)
*D*_*I*_	Infection distance	0.005	0.005
*μ*_*e*_	Exposed time mean	11.6^a^	11.6^a^
*σ*_*e*_	Exposed time standard deviation	1.9^a^	1.9^a^
*μ*_*i*_	Infection time mean	18.49^a^	18.49^a^
*σ*_*i*_	Infection time standard deviation	3.71^a^	3.71^a^
[*M*_1_, *M*_2_]	Mobility uniform prior range	[0.005, 0.025]	[0.005, 0.025]
[*J*_1_, *J*_2_]	Jumping probability uniform prior range	[0, 0]	[0, 0.001]

^*a*^ Consistent with incubation and infection period (from fever to recovery) of smallpox as reported in [31], if the unit of time is days.

For reference, at the lowest possible mobility value of 0.005, an agent can, at most, move across a 0.005 fraction of the domain length in one time step of length Δ*t* = 0.1 (Table 1). At the highest mobility value, an agent can, at most, move across a 0.025 fraction of the domain length (Table 1).

The range of mobilities and jumping probabilities was chosen with respect to the fixed parameters (including domain size, distance of infection, and total number of agents) such that there was significant variation in disease spread across mobility and jumping probability values. Fixed parameters (domain boundaries, distance of infection, total number of agents, and length of exposure and infection) were chosen such that in combination, they would not lead to an extremely dense or extremely sparse population. For example, if the chosen domain size, distance of infection, and number of agents caused all agents to be in constant “contact” (within distance of infection of other agents) or to almost never be in “contact” with other agents, the effects of mobility and jumping probability variation would be minimal and the parameters we wish to calibrate would be non-identifiable.

### Bayesian inference

The Bayes theorem, shown in Eq 2, is used to update prior beliefs about the parameters of a model *θ* using some data *y* to arrive at a posterior distribution over the parameters [32]. The prior distribution is denoted as *p*(*θ*) and the posterior distribution is denoted by *p*(*θ*|*y*). The likelihood *p*(*y*|*θ*) describes the probability of data *y* given parameters *θ*, evaluated as a function of *θ* for some constant *y*. If it is instead taken as a function of *y* with a constant *θ*, *p*(*y*|*θ*) is a probability density function of obtaining data *y* when a stochastic model is run with parameters *θ*. The prior knowledge about the distribution of parameters *θ* can be based on expert knowledge or be “uninformative” (often a diffuse distribution).

$$\begin{array}{c} p(\theta |y)\propto p(y|\theta )p(\theta ) \end{array}$$ (2)

#### Calibration method 1: Bayesian inference using an empirical likelihood function and MCMC

MCMC methods follow a parameter sampling procedure designed to converge to the posterior distribution over many repetitions [33]. This is a natural fit to the SBC procedure, as the outputs of MCMC are posterior samples. MCMC is commonly used to perform Bayesian inference, as it provides a flexible numeric approximation scheme that is well-suited to complex likelihood functions, like those that commonly arise in disease spread modeling.

We use an adaptive Metropolis MCMC (AMCMC) algorithm [34] to determine the posterior distribution of the parameters given an observed data set—in particular, we use the implementation from the package UQTk [35].

The initial chain location for the one-parameter case was *θ*_0_ = (*M* = 0.0151), while the initial chain location for the two-parameter case was *θ*_0_ = (*M* = 0.0151, *J* = 0.00051). The initial chain location was the same for all trials. AMCMC is run for 75*K* iterations, with a non-adaptive period length *ν* = 5000 and a burn-in of 25*K* iterations. Further details are described in the supplementary information (S2 Appendix).

#### Prior and likelihood construction

We use a uniform prior distribution for all parameters. The uniform distribution ranges for the parameters in the one- and two-parameter cases are in Table 1.

**Fig 5:**
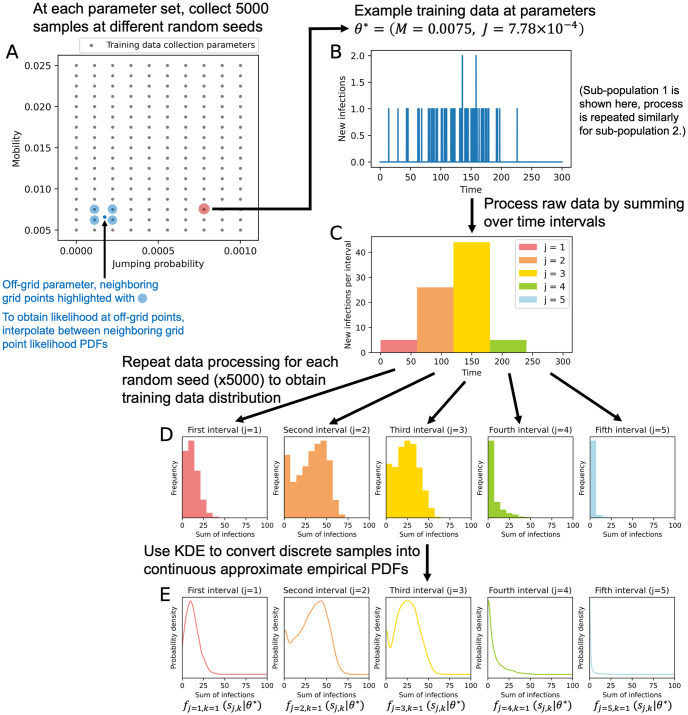
Diagram of approximate empirical PDF creation in the two-parameter case for sub-population 1. (A) Training data sampling points. (B) Example training data sample. (C) Processed training data sample. (D) For each time interval, a histogram of total new infections across many samples. (E) Approximate empirical PDFs of total new infections for a given time interval, sub-population, and parameter set. The one-parameter case follows a similar process with jumping probability held constant at *J* = 0.

At each MCMC step, we must evaluate the likelihood, *p*(*x*|*θ*_*i*_), for a given data sample and parameter set. Since the likelihood cannot be analytically determined, we instead use approximate empirical probability density functions (PDFs) constructed from training data. As training data samples are distributed according to $\tilde{x}∼p\left(x|\theta \right)$, a sufficient number of training data samples should allow for a reasonable approximation of the empirical PDF at a fixed parameter value. Then, these values can be used to determine the likelihood. The process of constructing these empirical PDFs is illustrated in Fig 5 and described in more detail below. Since this training data is collected in advance, the model does not need to be run during calibration to collect likelihood values, which helps reduce computational time.

The approximate empirical PDFs were made using a training data set sampled along a Cartesian grid of parameter values. Mobility *M* was sampled at 17 equally spaced points between [0.005, 0.025]. For the one-parameter case, the jumping probability *J* was held constant at 0, while for the two-parameter case, jumping probability was sampled at 10 equally spaced points between [0, 0.001] (Fig 5A). At each parameter combination, we ran 5000 simulations with different random seeds. Each training data sample, $\tilde{x}$, contains a time series of new infections per time step for each sub-population. We represent the number of new infections at time step *t* and for sub-population *k* as ${\tilde{x}}_{t,k}$.

The small population sizes and time steps in the simulations result in high-dimensional, sparse data (Fig 5B). When unprocessed, it is difficult to summarize this data into meaningful likelihoods. Therefore, each vector of infections over time for a single sub-population, ${\tilde{x}}_{:,k}$, was processed by summing the total number of new infections over *n* = 5 evenly spaced time intervals, resulting in a training data set of summary statistics $\tilde{s}$ containing new infection counts ${\tilde{s}}_{j,k}$, where *j* = 1, …, 5 is the time interval (Fig 5C). Likewise, test data time series **x**_:,**k**_ are summed over the same time intervals to result in test data summary statistics, *s*_*j*,*k*_ (where “test data” refers to a dataset for which we wish to estimate parameters). By binning the data in this way, we greatly reduce the dimensionality of the data and improve interpretability.

**Fig 6:**
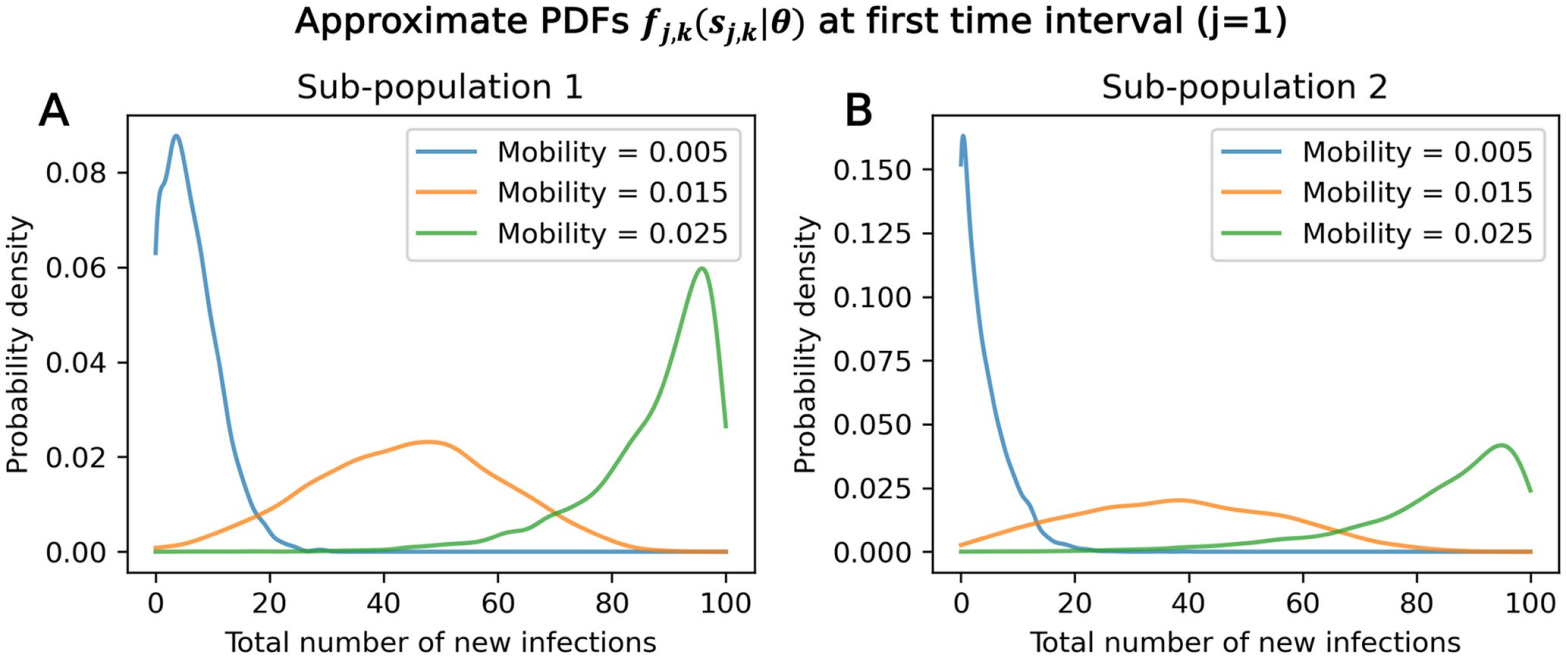
Approximate empirical PDFs. Approximate empirical PDFs for sub-population 1 (A) and 2 (B) at the first time interval generated via KDE from training data. Jumping probability is constant at 7.78 × 10^−4^.

Once training data is collected and processed into summary statistics, we convert these discrete samples (Fig 5D) into continuous PDFs using Gaussian kernel density estimates (KDEs) [36] (Fig 5E). Specifically, we create PDFs (*f*_*j*,*k*_(*s*_*j*,*k*_|*θ*)) of new infections in time interval *j* and sub-population *k* for a given fixed parameter set using KDE on the training data. These PDFs describe the approximate empirical probability of obtaining a certain number of new infections at a specified time interval and sub-population, and for a specified parameter set. Additional examples are shown in Fig 6: Fig 6A shows, at time interval *j* = 1 and sub-population *k* = 1, how the approximate empirical PDFs change for different mobility values. At low mobility values, where there is less movement and slower disease spread, a smaller number of infections is more likely in the first time interval than at higher mobility values.

For a given parameter set, there are *n* × *m* PDFs, where *n* is the number of time intervals, and *m* is the number of sub-populations. To expand beyond the discrete parameter combinations contained in the training data set, PDFs can be interpolated from neighboring training data parameter PDFs (Fig 5A) [37]. The interpolation process is described in detail in the S1 Appendix.

Given a test data sample *x* and corresponding summary statistics *s*, MCMC is used to sample the posterior, *p*(*θ*|*s*). For each sample *θ*_*i*_, where *i* is the sample ID, the log-likelihood *l*(*s*|*θ*_*i*_) is evaluated according to Eq 3 using the empirical PDFs, *f*_*j*,*k*_(*s*_*j*,*k*_|*θ*). This construction assumes that data is independent across time intervals and sub-populations.

$$\begin{array}{c} l\left(s|\theta_{i}\right)=\sum_{k=1}^{m}\sum_{j=1}^{n}log\left(f_{j,k}\left(s_{j,k}|\theta_{i}\right)\right) \end{array}$$ (3)

Runtime for calibration method 1 on a single test data set takes around an hour in the one-parameter case, and multiple hours in the two-parameter case when run on a laptop.

#### Calibration method 2: Likelihood-free Bayesian inference with an ABC algorithm

ABC methods allow one to avoid direct likelihood calculations in favor of a chosen distance function, ‖ ⋅ ‖. In an ABC rejection algorithm, parameters are sampled from the prior, *θ*_*i*_ ∼ *p*(*θ*), and run through the model, outputting training data $\tilde{x}$ [38]. The data are processed to result in summary statistics $\tilde{s}$ or *s* (training data summary statistics and observed test data summary statistics, respectively), generally chosen to encapsulate important information while reducing dimensionality. Samples are weighted according to a weight function, *K*_*ϵ*_, in which “closer” matches to the summary statistics of observed test data *s* are assigned higher weights, while poor matches are assigned small or zero weights. *ϵ* is a tolerance proxy parameter controlling the range of accepted samples. This process results in weighted samples distributed along $p_{\epsilon }\left(\theta |s\right)=\int p_{\epsilon }\left(\theta ,\tilde{s}|s\right)d\tilde{s}$, which is approximately equal to the true desired posterior, *p*(*θ*|*x*) [39, 40].

$$\begin{array}{c} p_{\epsilon }\left(\theta ,\tilde{s}|s\right)\propto K_{\epsilon }\left(\right\|\tilde{s},s\left\|\right)p\left(\tilde{s}|\theta \right)p\left(\theta \right) \end{array}$$ (4)

This algorithm formalizes the intuitive idea that similar data sets are often able to be generated from similar parameters, allowing us to infer the parameters of observed data by finding similar simulated runs. Qualitatively, we expect that if good matches are found with data from a wide range of parameters, then the posterior will be wide and uncertainty will be large, and vice versa.

**Fig 7:**
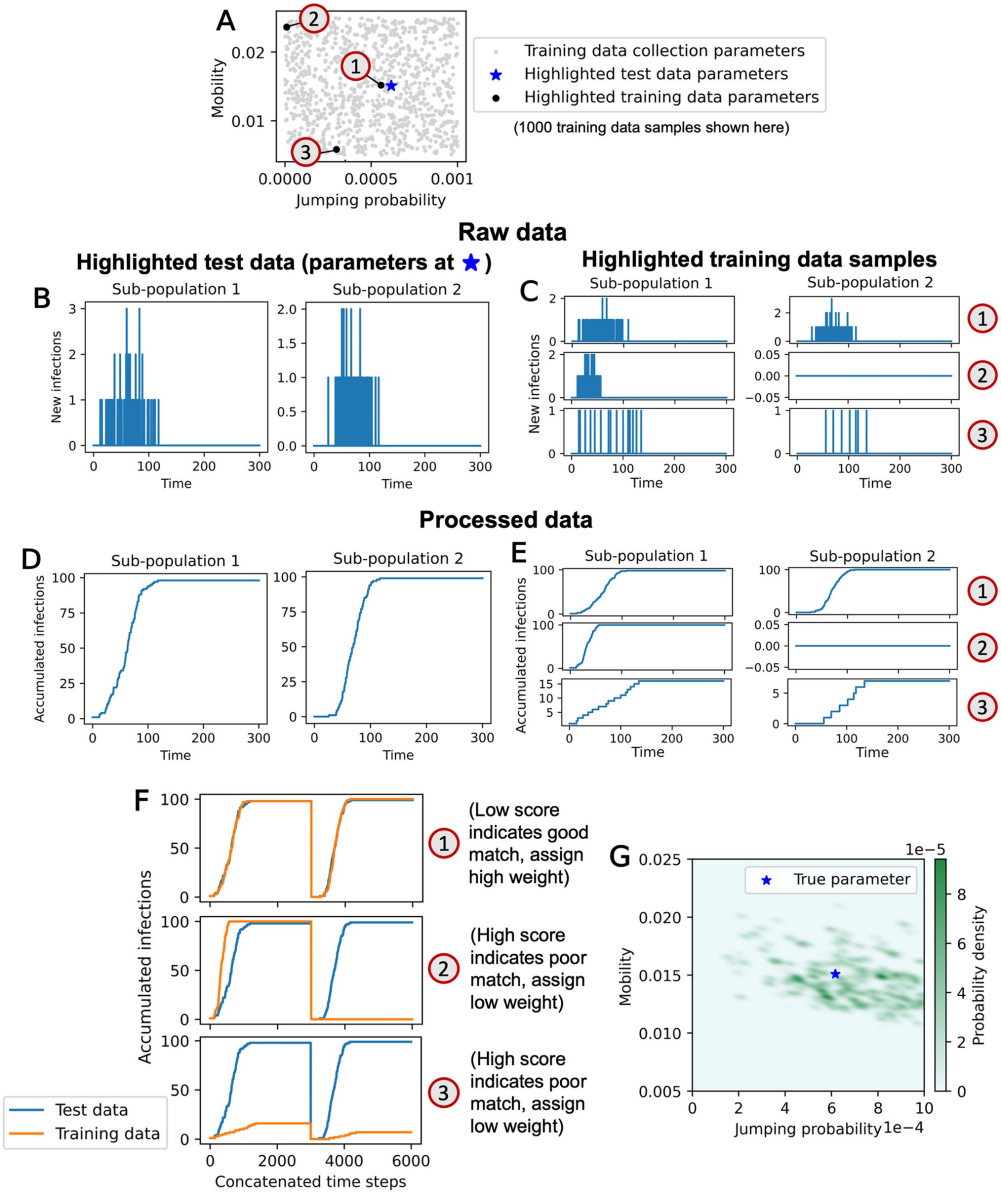
Diagram of ABC rejection algorithm process in two-parameter case. (A) Training data sampling points and example test data point. (B) Example test data sample. (C) Example training data samples. (D) Processed test data sample. (E) Processed training data samples. (F) Visualization of scoring process. (G) Resulting posterior from calibration process. The one-parameter case process is similar, but with jumping probability held constant at *J* = 0 and only one sub-population.

We create a training data set of samples $\tilde{x}$ with parameter values sampled from the priors as specified in Table 1 (two-parameter case shown in Fig 7A). A total of 85K training runs were generated for both the one-parameter and two-parameter cases. Within the two training data sets, each run used a different random seed. As before, the training data consists of time series of new infections per time step.

To generate summary statistics *s*, we accumulate the raw data (Fig 7B and 7C) over time, such that $s_{t,k}=\sum_{i=1}^{t}x_{i,k}$ (Fig 7D and 7E). This allows all the information in the original data set to be preserved (unlike in a binning process), which means our approximate posterior *p*_*ϵ*_(*θ*|*s*) is exactly equal to *p*_*ϵ*_(*θ*|*x*). This process was chosen after running preliminary trials using summary statistics generated by summing total infections over a sliding window of variable size, which found that larger windows worked better. Accumulation over time (our chosen summary statistic process) effectively sums over a sliding window using the largest possible window size.

In general, appropriate summary statistic choice depends on the specific problem—a variety of strategies have been used in previous work, from simply using common metrics in the field, trialing many sets of plausible summary statistics [41], attempting to achieve “approximate statistical sufficiency” by iteratively testing the effect of additional statistic components [42], or by using auxiliary likelihoods to summarize the data into, for example, a maximum likelihood estimator [43]. Further review of the techniques for developing summary statistics can be found in [41, 44, 45].

Our summary statistic generation process does not decrease the dimensionality of the data, which most ABC implementations aim to do when creating summary statistics in order to combat low computational efficiency due to increasing difficulty in finding close matches. We instead avoid low efficiency by using a relative distance function, as described below.

To define our distance function, we first establish scores: In the one-parameter case, scores are defined using an *L*_2_ norm: $\rho \left(\tilde{s},s\right)={\left\|{\tilde{s}}_{:,1}-s_{:,1}\right\|}_{2}$. In the two-parameter case, the data from sub-populations 1 and 2 are concatenated into a vector $\left({\tilde{s}}_{:,1},{\tilde{s}}_{:,2}\right)$. The score is then calculated: $\rho \left(\tilde{s},s\right)={\left\|\left({\tilde{s}}_{:,1},{\tilde{s}}_{:,2}\right)-\left(s_{:,1},s_{:,2}\right)\right\|}_{2}$. Then, the distance function, ‖ ⋅ ‖, is the sample score rank statistic relative to the rest of the training data. For a training data set consisting of $\left\{{\tilde{s}}^{0},{\tilde{s}}^{1},...,{\tilde{s}}^{N}\right\}$, the training data sample ${\tilde{s}}^{q}$ with the lowest score $\rho ({\tilde{s}}^{q},s)$ relative to other training data samples has rank statistic 0, therefore its distance function is evaluated: $\|{\tilde{s}}^{q},s\|=rank\left(\left\{\rho \left({\tilde{s}}^{0},s\right),...,\rho \left({\tilde{s}}^{N},s\right)\right\}\backslash \left\{\rho \left({\tilde{s}}^{q},s\right)\right\},\rho \left({\tilde{s}}^{q},s\right)\right)=0$. A visualization of scoring in the two-parameter case is shown in Fig 7F.

**Fig 8:**
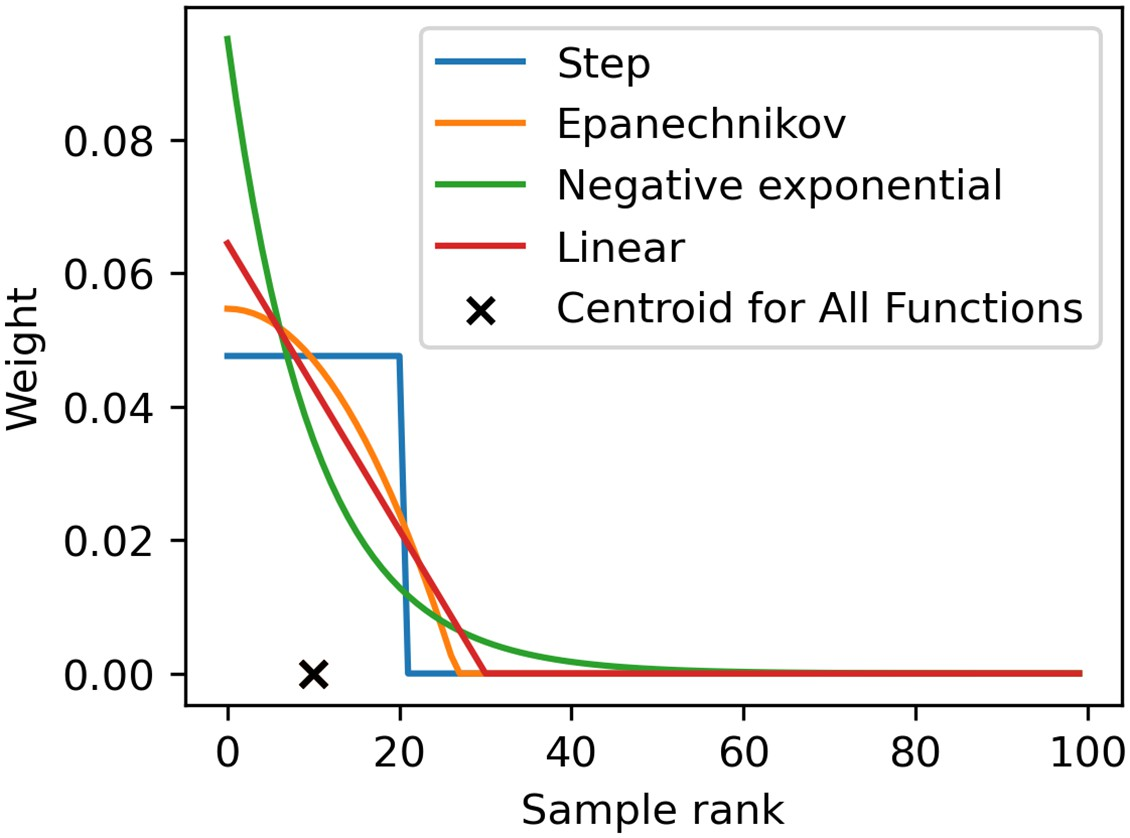
Weight functions. Weight functions with step, linear, Epanechnikov, and negative exponential shapes. Normalized by the number of training data samples (100), the centroid value is *ϵ*/100 = 0.1.

We test four weight functions, shown in Fig 8 and Eq 5 (where *H*() is the Heavyside function). The step function scaled by a normalizing constant *c* leads to an accept-reject algorithm with no intermediate weighting. The other three weight functions provide some level of smoothing: the Epanechnikov kernel, a negative exponential, and a linear piece-wise function. The linear function and Epanechnikov kernel both have hard cut-offs, above which samples are rejected (weight is set to 0), while the negative exponential places some non-zero weight on every sample.

$$\begin{array}{c} K_{\epsilon }\left(d\right)=\{\begin{array}{cc} cH(\delta_{\epsilon }-d) & \;\text{Step}\\ c\left(\delta_{\epsilon }-d\right)H\left(\delta_{\epsilon }-d\right) & \;\text{Linear}\\ c\delta_{\epsilon }^{-1}\left(1-{\left(d/\delta_{\epsilon }\right)}^{2}\right)H\left(\delta_{\epsilon }-d\right) & \;\text{Epanechnikov}\\ c\delta_{\epsilon }^{-d} & \;\text{Negative}\;\text{exponential} \end{array} \end{array}$$ (5)

Each weight function contains a variable *δ*_*ϵ*_, defined as the value at which the centroid of the weight function *K*_*ϵ*_(*d*) over positive values *d* = [0, ∞) is equal to *ϵ*. Because the distance measures are calculated using rank statistics, *ϵ* determines the fixed proportion of samples which will be assigned non-zero weights according to the weight function used—smaller centroid values assign non-zero weights only to samples with the smallest distance measures and vice versa. Using *ϵ* to control the centroid allows us to directly compare weight function shapes while holding the tolerance to an equivalent standard. Here, the centroid, *ϵ*, controls a balance between accuracy (smaller *ϵ* means accepted samples are more similar to the test data) and computational speed (larger *ϵ* means a higher ratio of accepted to not-accepted samples) [46]. Previous work has established some convergence properties as *ϵ* → 0, though this is still a developing area of research [46, 47]. As long as *ϵ* is sufficiently small, we expect a reasonable posterior approximation [48]. To determine an appropriate *ϵ* value, we ran a hyperparameter sweep on *ϵ* along with two other hyperparameters (weight function shapes and KDE bandwidth values) in the one-parameter case, which is discussed further in the Results section.

Algorithm 4 shows pseudocode of our implementation of ABC, which pulls weighted samples (*θ*_*i*_, *w*_*i*_) of the approximate posterior *p*_*ϵ*_(*θ*|*x*) given observed data *x*. The process of converting observed data *x* into summary statistics *s* is represented as *s* = *g*(*x*).

**Algorithm 4** ABC algorithm

1: Initialize empty list of weighted approximate posterior samples, *θ*_*ABC*_

2: Initialize empty list, thetas

3: Initialize empty list, scores

4: **for** i = 0 to n **do**

5:  *θ*_*i*_ ∼ *p*(*θ*)

6:  $\tilde{x}∼p\left(x|\theta_{i}\right)$

7:  $\tilde{s}=g\left(\tilde{x}\right)$

8:  $\text{score}=\rho (\tilde{s},s)$

9:  Append score to scores

10:  thetas[i] = *θ*_*i*_

11: **end for**

12: **for** i = 0 to n **do**

13:  *d* = *rank*(scores, scores[i])

14:  *θ*_*i*_ = thetas[i]

15:  *w*_*i*_ = *K*_*ϵ*_(*d*)

16:  Append (*θ*_*i*_, *w*_*i*_) to *θ*_*ABC*_

17: **end for**

The weighted samples from the ABC rejection algorithm are then converted into an approximate posterior using Gaussian KDE. To control the level of smoothing, the covariance matrix of the input data is scaled by the squared KDE bandwidth to obtain the kernel covariance matrix. Larger KDE bandwidth values lead to higher smoothing, and vice versa. Contributions to the total PDF are scaled according to a training data point’s assigned weight—in particular, the total posterior integral contribution of a point is scaled to be proportional to the weight. If the weights instead simply scaled Gaussian PDF values through direct multiplication, training points near the edge of the prior domain would become increasingly underweighted when bandwidths are high, since a significant portion of the individual Gaussian probability density would lie outside the prior domain and therefore not have positive posterior density.

Runtime for calibration method 2 varies depending on hyperparameters. Calibration to a single test data set takes a couple seconds in the one-parameter case, and between a couple seconds and a couple minutes in the two-parameter case on a laptop.

#### Testing inference performance

In accordance with simulation-based calibration testing procedures, test data was generated by pulling parameter values from the prior (priors specified in Table 1), then running the model with those parameters. 1000 model runs were collected for the one-parameter case test data set, and 1666 model runs were collected for the two-parameter case test data set.

We then use simulation-based calibration to test the performance of Bayesian inference. For the first calibration method using empirical likelihoods and MCMC, we follow the thinning and truncation procedure described in Talts et al. [24] with a slight alteration: if the chain length after thinning, *Q*, is less than *L*, we approximate the rank statistic according to Eq 6,

$$\begin{array}{c} rank\left(\left\{\theta_{1},...,\theta_{L}\right\},\tilde{\theta }\right)\approx round\left(\left(L/Q\right)\cdot \sum_{i=1}^{Q}1\left(\tilde{\theta }>\theta_{i}\right)\right) \end{array}$$ (6)

where *round* returns the nearest integer. This is an approximation, under which uniformity of ranks is no longer fully guaranteed for correct posterior inference. We also adhere to their suggestion to thin multi-variate parameter chains once based on the smallest effective sample size (ESS) identified over the parameter components. We calculate the ESS using PyUQTk [35].

For the second calibration method using ABC, the variable weighting makes it difficult to directly pull samples from the posterior, as is done with MCMC. Instead, we use the continuous posterior constructed with KDE to determine the exact quantiles of the test data samples with respect to the predicted posterior. For a correct inference, the quantile values are expected to be *continuously* uniformly distributed.

We additionally compare the performance of ABC across different hyperparameters using the continuous ranked probability score (CRPS) [23]. The CRPS evaluates the relationship between a sample and a cumulative distribution function (CDF) in order to judge whether that sample was drawn from the distribution defined by the CDF. Here, we wish to determine whether the parameter sample $\tilde{\theta }$ is drawn from the distribution of our inferred posterior, $p(\theta |\tilde{y})$. The CRPS is defined such that its expected value is minimized if the sample is indeed drawn from the given distribution. It summarizes both calibration (the statistical consistency tested with SBC) as well as “sharpness” (the specificity of the predicted posteriors, not addressed by SBC). As uniform SBC histograms are a necessary but not sufficient condition for correct posterior inference procedures, CRPS provides a way of differentiating between two methods which both “pass” under SBC diagnostics. CRPS is defined in Eq 7, where *F* is the posterior PDF, $p(\theta |\tilde{y})$, converted into a CDF [23].

$$\begin{array}{c} CRPS\left(F,\tilde{\theta }\right)=\int_{-\infty }^{\infty }{\left(F\left(y\right)-1\left(y\geq \tilde{\theta }\right)\right)}^{2}dy \end{array}$$ (7)

## Results

### Calibration method 1: Bayesian inference using MCMC

**Fig 9:**
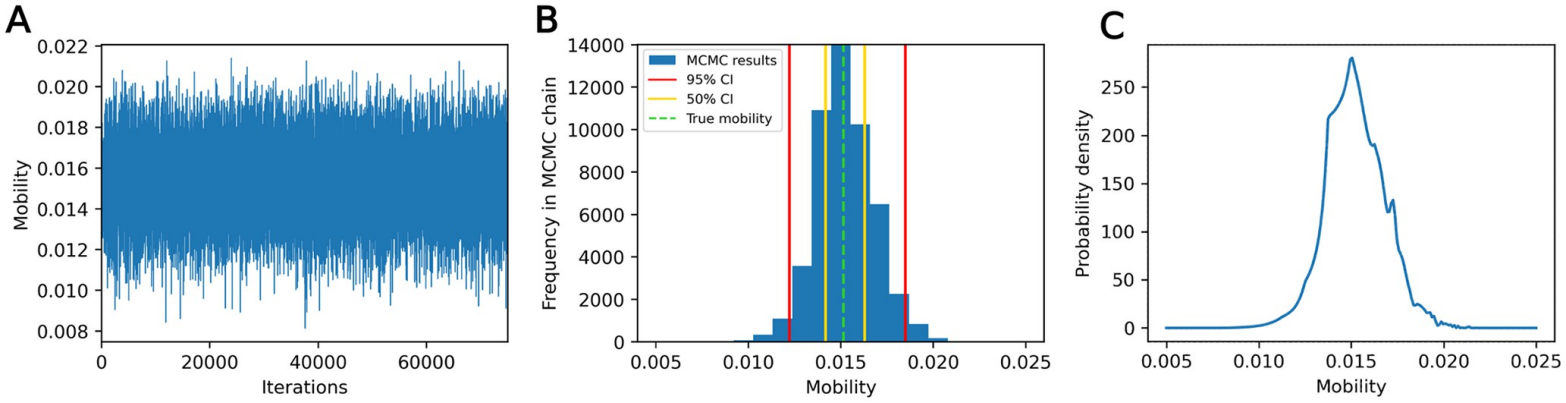
Example results from calibration method 1 in the one-parameter case. (A) MCMC trace plot. (B) Histogram of MCMC chain results, with 50% and 95% credible interval bounds marked. (C) Posterior function sampled over grid of mobility values.

**Fig 10:**
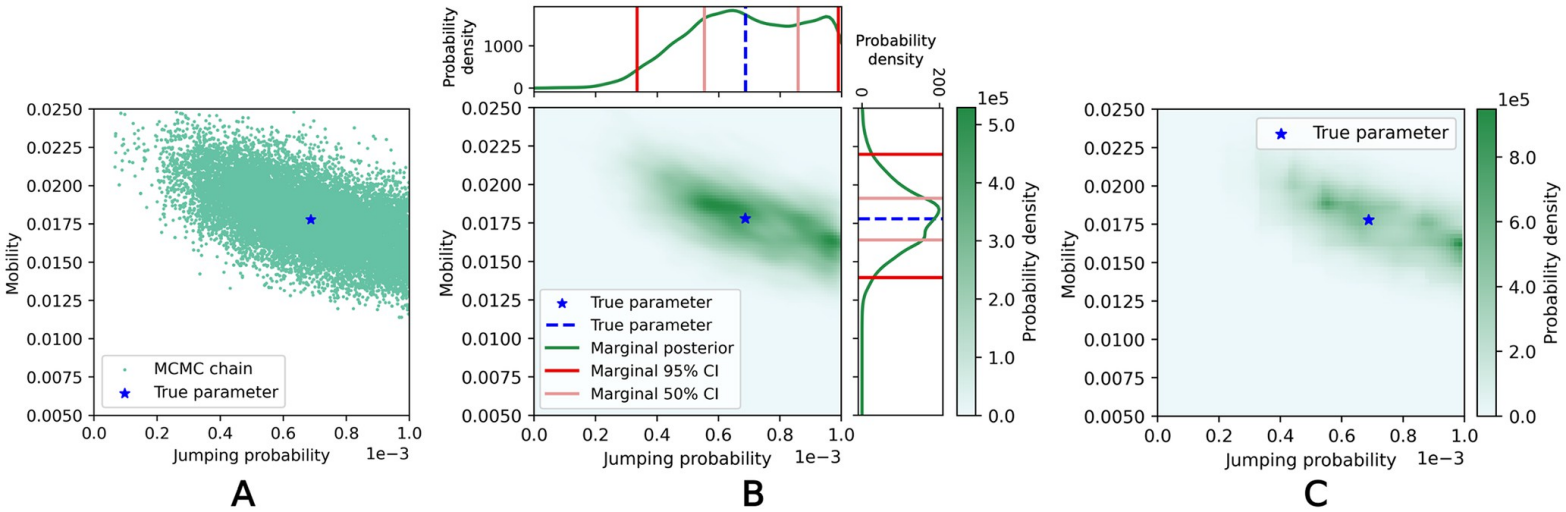
Example results from calibration method 1 in the two-parameter case. (A) Scatter plot of MCMC chain values. (B) Approximate posterior constructed using KDE, with 50% and 95% credible interval bounds marked. (C) Posterior function sampled over grid of parameter values.

Since a uniform prior was used in generating the posterior, a brute-force grid sampling of the likelihood function (Eq 3) can be used to check for MCMC convergence, as the likelihood should be proportional to the posterior over the prior support. By rescaling the likelihood, we obtain the grid-sampled posterior. Qualitatively, nearly all MCMC-constructed posteriors appeared to match the shape of the corresponding grid-sampled posterior, which indicates sufficient MCMC iterations (see examples in Figs 9 and 10, outlier cases are discussed later). For the one-parameter case, the ESS had a mean value of 10642. For the two-parameter case, the ESS for mobility values had a mean of 5201, while the ESS for jumping probability values had a mean of 5191. These ESS values also suggest sufficient iterations to reach convergence.

Example calibration results are shown in Figs 9 and 10 with 50% and 95% credible intervals highlighted for the one-parameter case and the marginal credible intervals highlighted in the two-parameter case. Recall that if a Bayesian inference analysis is performed correctly, the “true” parameter value, $\tilde{\theta }$, is expected to fall within the 50% credible interval at a 50% rate across test data samples (given that test data is drawn according to $\tilde{y}∼p\left(y|\tilde{\theta }\right)$), and likewise for the 95% interval [20, 24]. This property only applies to one parameter dimension at a time—for a multivariate parameter set, a component of the “true” parameter $\tilde{\theta }$ is expected to fall within the 50% *marginal* credible interval at a 50% rate across test data samples.

**Fig 11:**
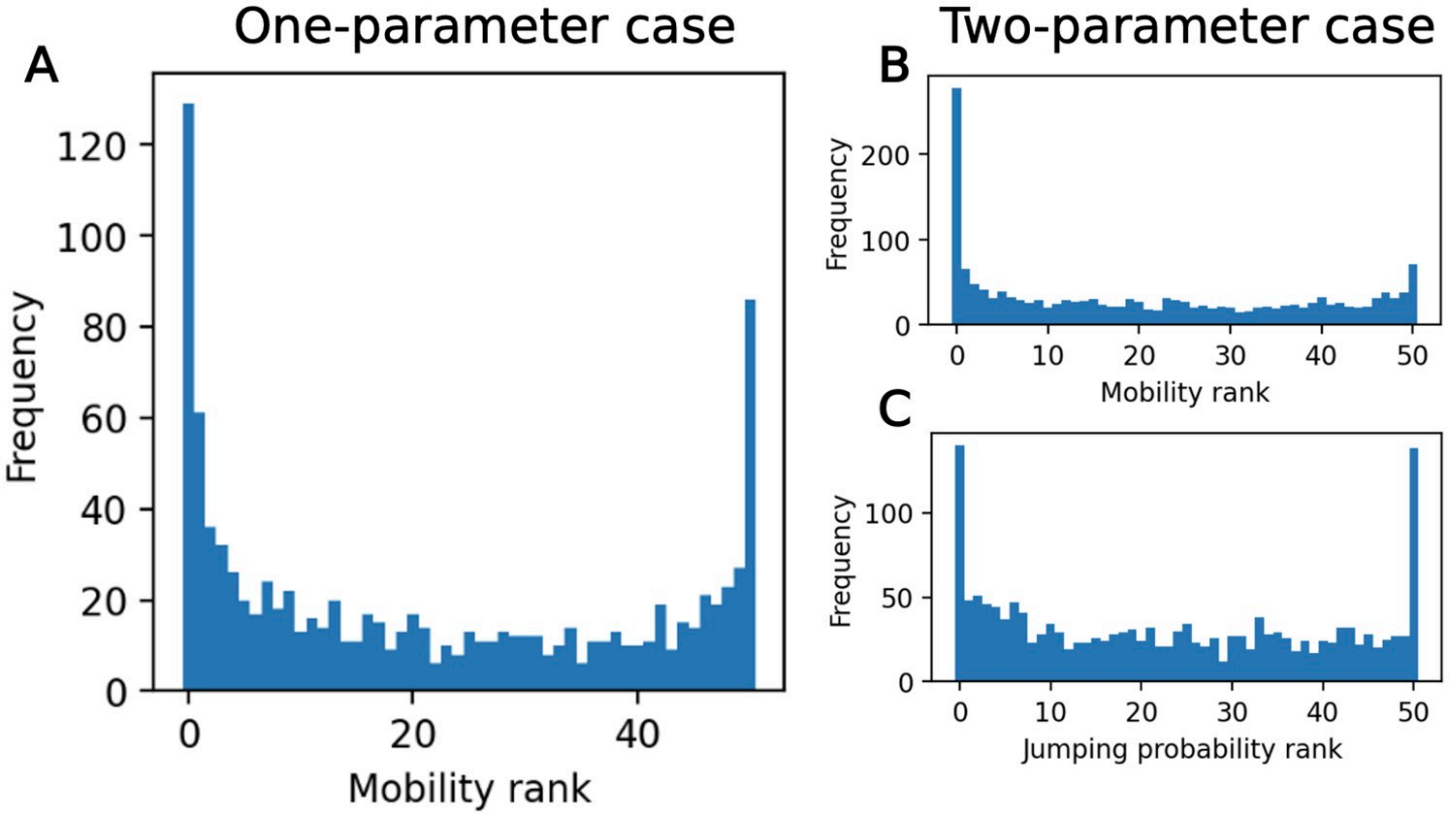
SBC results for calibration method 1. (A) One-parameter case results over the mobility parameter. (B) Two-parameter case results over the mobility parameter. (C) Two-parameter case results over the jumping probability parameter.

The rank histograms from simulation-based calibration are shown in Fig 11, with *L* = 50. For both the one- and two-parameter cases, the ranks are non-uniform, with high frequencies of low and high ranks. In the one-parameter case, none of the samples had thinned chains with length shorter than *L*, while in the two-parameter case, 6.9% had length shorter than *L*—this could potentially contribute to the higher frequencies of low and high ranks. However, we expect that these non-uniform effects would likely persist even if these samples were corrected through additional MCMC iterations. This is further justified in the S3 Appendix.

### Calibration method 2: Likelihood-free Bayesian inference with an ABC algorithm

**Fig 12:**
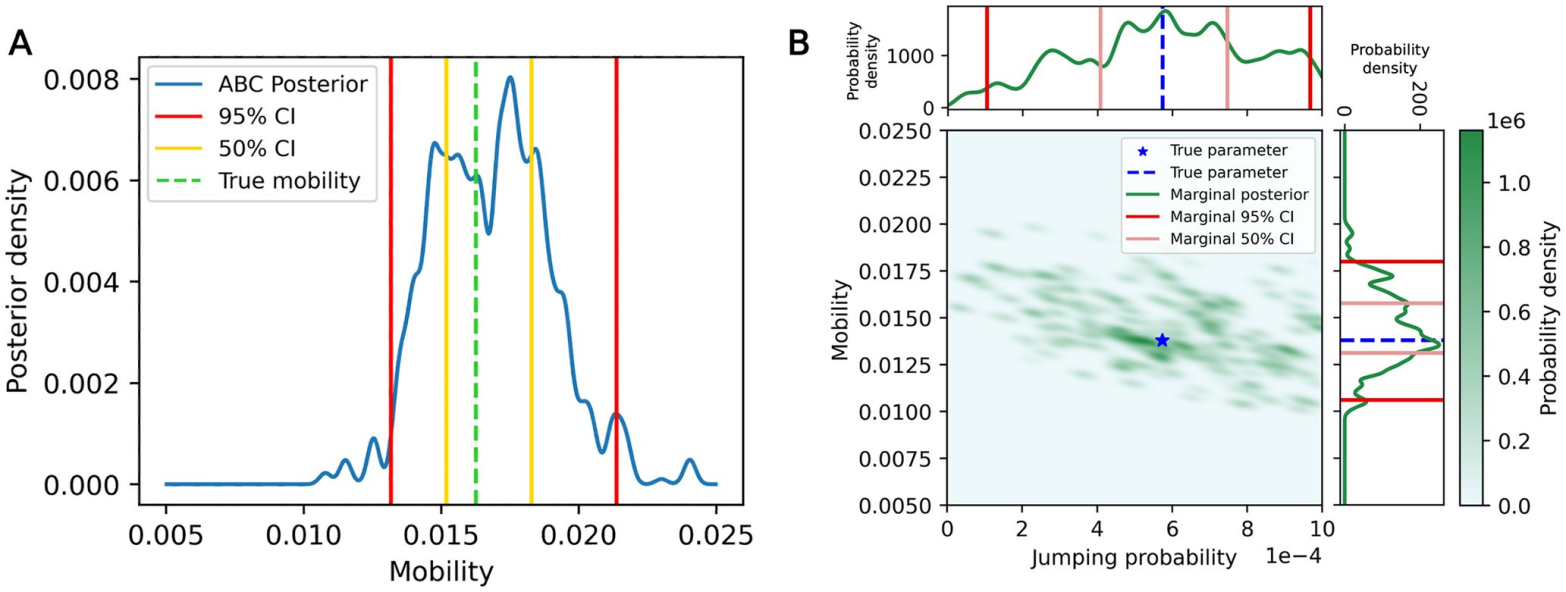
Examples posteriors generated by calibration method 2. (A) In the one-parameter case. (B) In the two-parameter case. 50% and 95% credible interval bounds and “true” parameter values are marked.

Example calibration results using the ABC rejection algorithm are shown in Fig 12, with 95% and 50% credible intervals (or marginal credible intervals, in the two-parameter case) highlighted. Higher KDE bandwidth and centroid values would both lead to a “smoother” appearance of the posterior from larger Gaussian kernels and more included data respectively.

There are three primary hyperparameters in the ABC process: the weight function shape, the weight function centroid value (*ϵ*), and the KDE bandwidth. To determine what effect these parameters have on calibration performance, we ran a hyperparameter sweep over the four weight function shapes (step, linear, negative exponential, and Epanechnikov), a range of centroid values (the values of *ϵ*, normalized by the number of training data points, were *ϵ*/85, 000 = [0.001, 0.01, 0.1, 1, 10]), and a range of bandwidth values (*BW* = [0.1, 0.3, 1, 3, 10]).

Note that for the step weight function, any training data $\tilde{s}$ with $rank(\rho \left(\tilde{s},s\right))\leq \epsilon *2$ will have equal non-zero weights. Since the maximum rank value in this case is 85,000, a normalized centroid value of *ϵ*/85, 000 = 1 or *ϵ*/85, 000 = 10 will both result in equal non-zero weights for all training data. Therefore, the results of ABC for the step weight function will be identical between normalized centroid values of 1 and 10.

**Fig 13:**
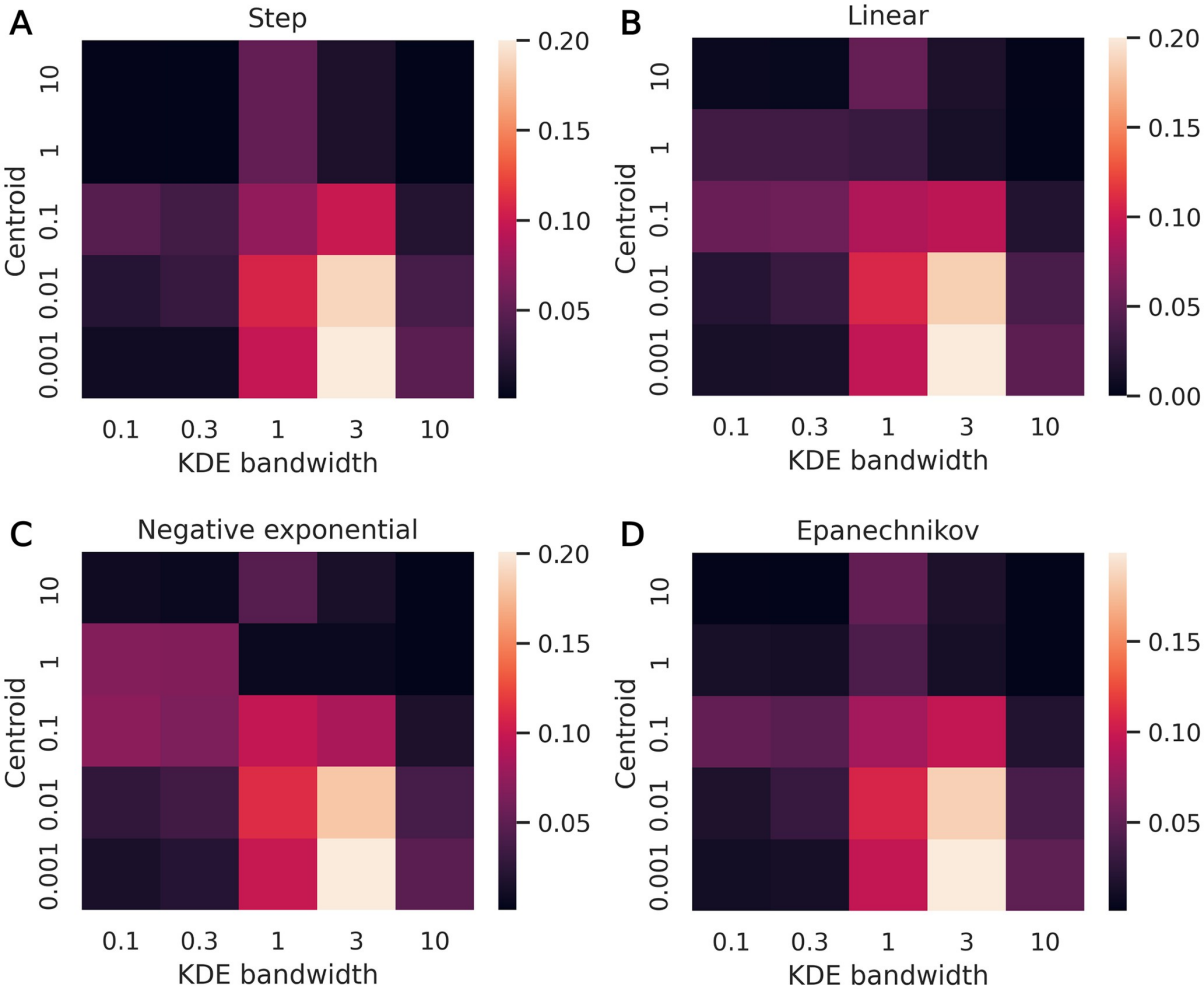
Calibration method 2 hyperparameter sweep results. The colorbar represents the absolute difference between 0.5 and the fraction of “true” mobility values that fell within their respective 50% credible intervals, shown across a range of KDE bandwidths and centroids. Weight function shapes are (A) step, (B) linear, (C) negative exponential, (D) Epanechnikov.

If the Bayesian inference is implemented correctly, we expect that the “true” parameter values ($\tilde{\theta }$) will fall within the 50% posterior credible interval at a 50% rate [20, 24]. Fig 13 shows the absolute difference between this expected rate, 0.5, and the observed rate of “true” mobility values falling within their respective 50% credible intervals. These values act as a weaker, but more easily visualized, proxy for the SBC histogram uniformity diagnostics. The same test data was used for each hyperparameter combination.

The absolute difference trends towards an increase, then decrease, as the KDE bandwidth increases. As KDE bandwidth increases, we expect the posterior to become more spread out, causing overdispersed posteriors—this is the likely cause of the increase in absolute difference. However, if overdispersed enough, the posteriors begin to resemble the uniform prior, which could cause the absolute differences to decrease at the highest bandwidth values. These overdispersed posteriors are able to satisfy SBC histogram uniformity because it is a necessary but not sufficient condition for correct inference procedures.

At smaller KDE bandwidth values, the absolute difference tends to increase, then decrease, as the centroid value increases. Similar to the trends with respect to the KDE bandwidth, this is likely due to higher centroid values leading to overdispersed posteriors. At higher KDE bandwidths, the absolute difference decreases with respect to centroid value, likely because the posteriors are already overdispersed even at low centroid values due to the high KDE bandwidths. Thereafter, further widening of the posterior due to increased centroid values is expected to push it towards a uniform prior, likely causing the observed decreases in the absolute difference. Absolute difference values are very similar across weight function shapes.

**Fig 14:**
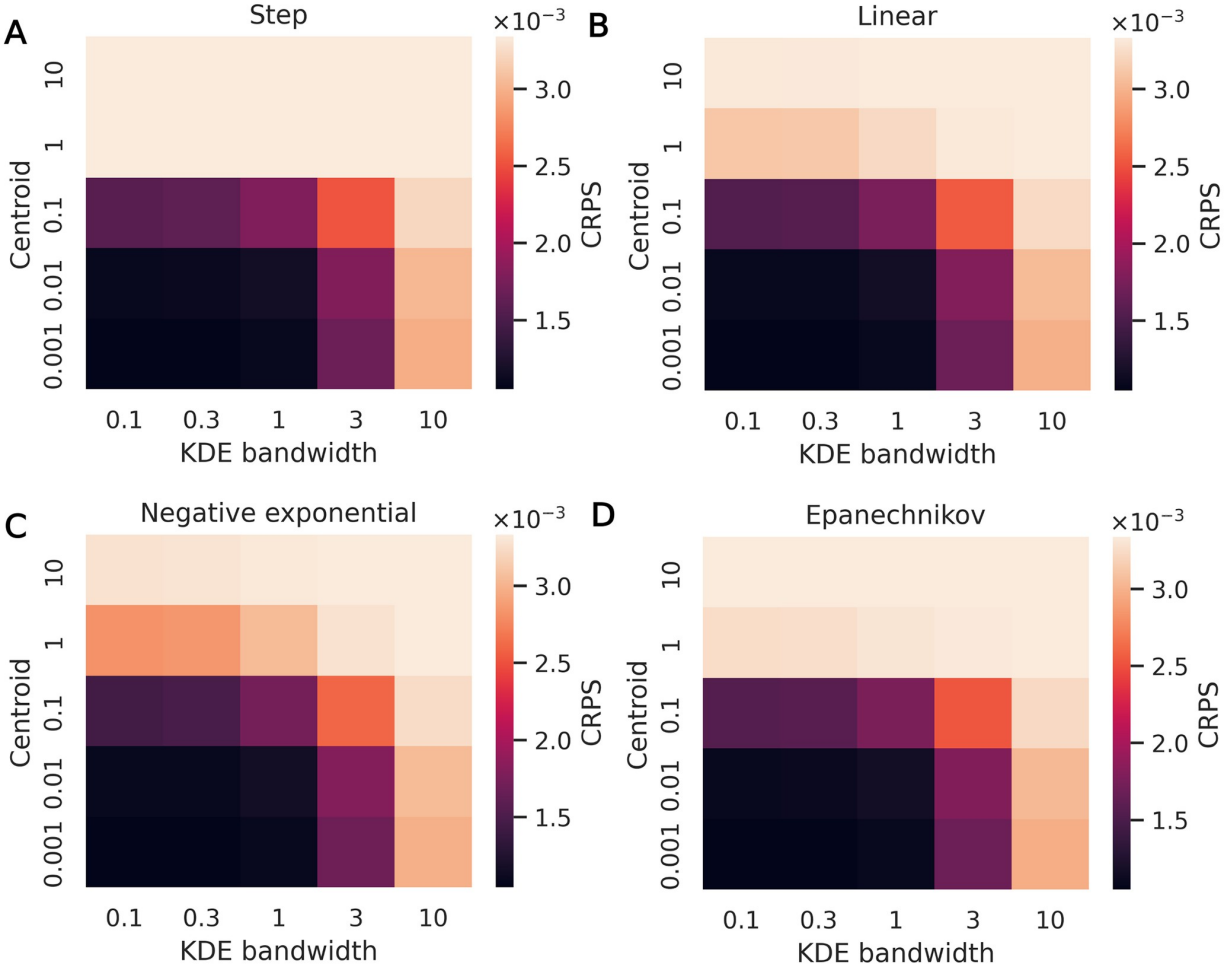
Calibration method 2 hyperparameter sweep results. Average CRPS for a range of KDE bandwidths and centroids (CRPS has the same unit as mobility, so it is unitless). Weight function shapes are (A) step, (B) linear, (C) negative exponential, (D) Epanechnikov.

We also evaluate the CRPS across hyperparameters, as shown in Fig 14. For each hyperparameter combination, the CRPS is averaged across the test data calibration runs. The resulting values act as a summary score, with lower scores indicating better statistical consistency between the posterior CDF and the test data as well as sharper CDF profiles, where the SBC procedure checks only the former. In Fig 14, CRPS values increase with both centroid and KDE bandwidth values. Where overdispered posterior predictions may begin to appear more calibrated (lower absolute differences in Fig 13) at high centroid and KDE bandwidth values, CRPS penalizes these posteriors distributions for being overdispersed and indicates better performance only at low centroid and KDE bandwidth values.

We run the two-parameter case with a normalized centroid value of 0.001 and a KDE bandwidth of 0.1, since those hyperparameters led to the best performance in the one-parameter hyperparameter sweep tests. As the weight function shape does not appear to have a significant impact on results, we use the Epanechnikov kernel, which combines varying weight values with the added computational benefit of setting many weights to zero, unlike the negative exponential function.

**Fig 15:**
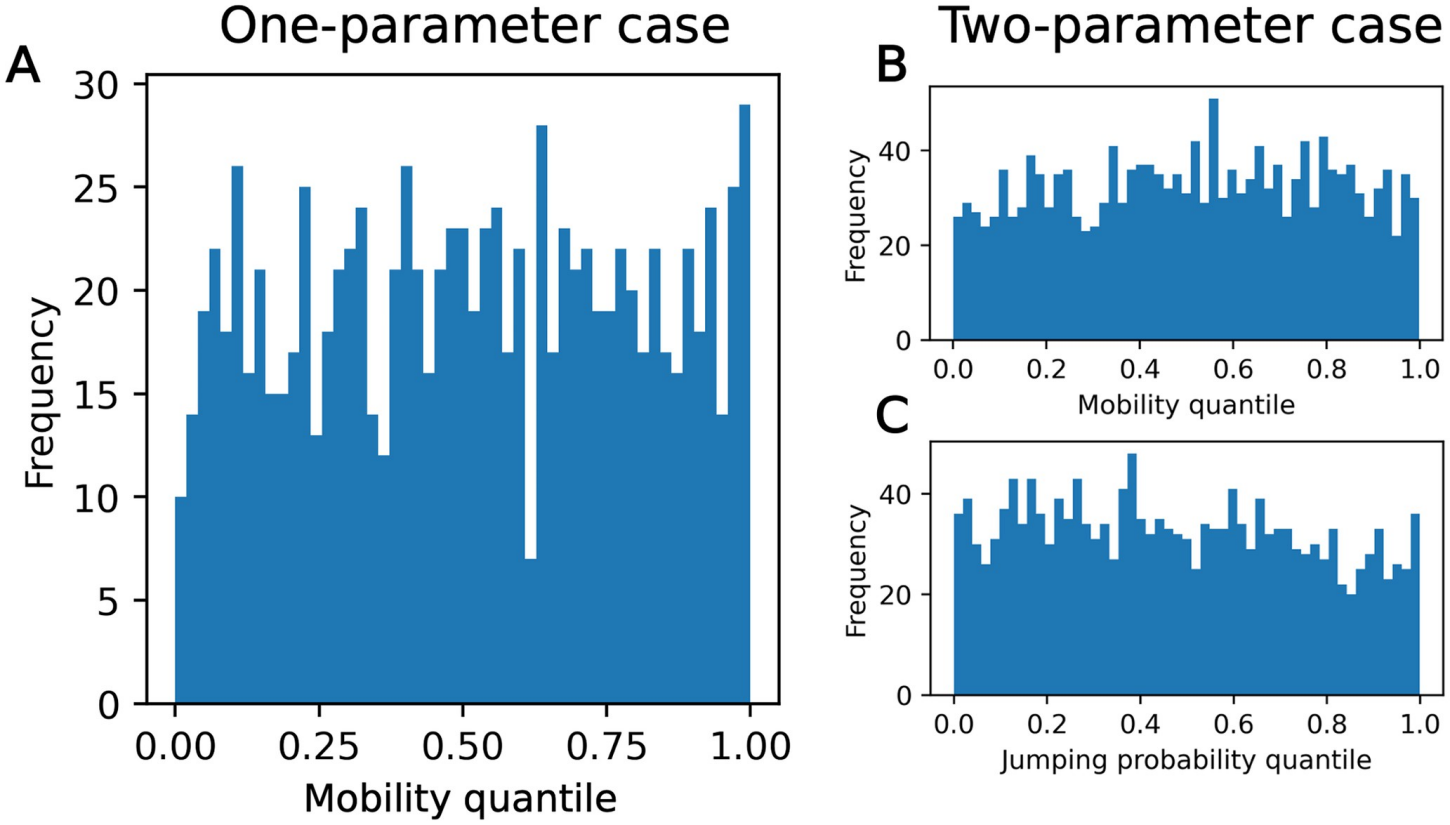
SBC results for calibration method 2. Produced with Epanechnikov shape function, normalized centroid of 0.001, and KDE bandwidth of 0.1. (A) One-parameter case results over the mobility parameter. (B) Two-parameter case results over the mobility parameter. (C) Two-parameter case results over the jumping probability parameter.

SBC rank histograms with 51 bins are shown in Fig 15. Visually, this calibration method using ABC rejection produced more uniform SBC histograms than calibration method 1 (Fig 15). A *χ*^2^ test returns a p-value of 0.383 for the one-parameter case, 0.314 for the mobility in the two-parameter case, and 0.404 for the jumping probability in the two-parameter case. The null hypothesis of uniformity cannot be rejected based on these p-values, as *p* > 0.05. While this is not a proof of uniformity, the p-values indicate that there is not statistically significant non-uniformity in these samples.

## Discussion

SBC histograms indicate that there are issues with calibration method 1, which uses an empirically-constructed likelihood function and MCMC sampling. In general, MCMC samples qualitatively appeared to converge to the grid-sampled posterior (Figs 9 and 10) and had high ESS, indicating no major computational issues in the MCMC process. We suggest, then, that these issues can be primarily attributed to the likelihood computation process, which contains several approximations and assumptions. First, there is some information lost when converting the raw data into summary statistics. There are also approximations in constructing the approximate empirical PDFs and in interpolating between these PDFs. Lastly, the likelihood computation assumes independence between time intervals, and for the two-parameter case, sub-populations. This assumption does not reflect the model set-up, since there is dependence between time intervals and sub-populations in the ABM. This assumption has been used previously in the literature [13, 15, 49]—in certain set-ups, the assumption acts as an approximate distance function, like in approximate Bayesian computation. However, in this case, it may have contributed to unfavorable aggregate results.

**Fig 16:**
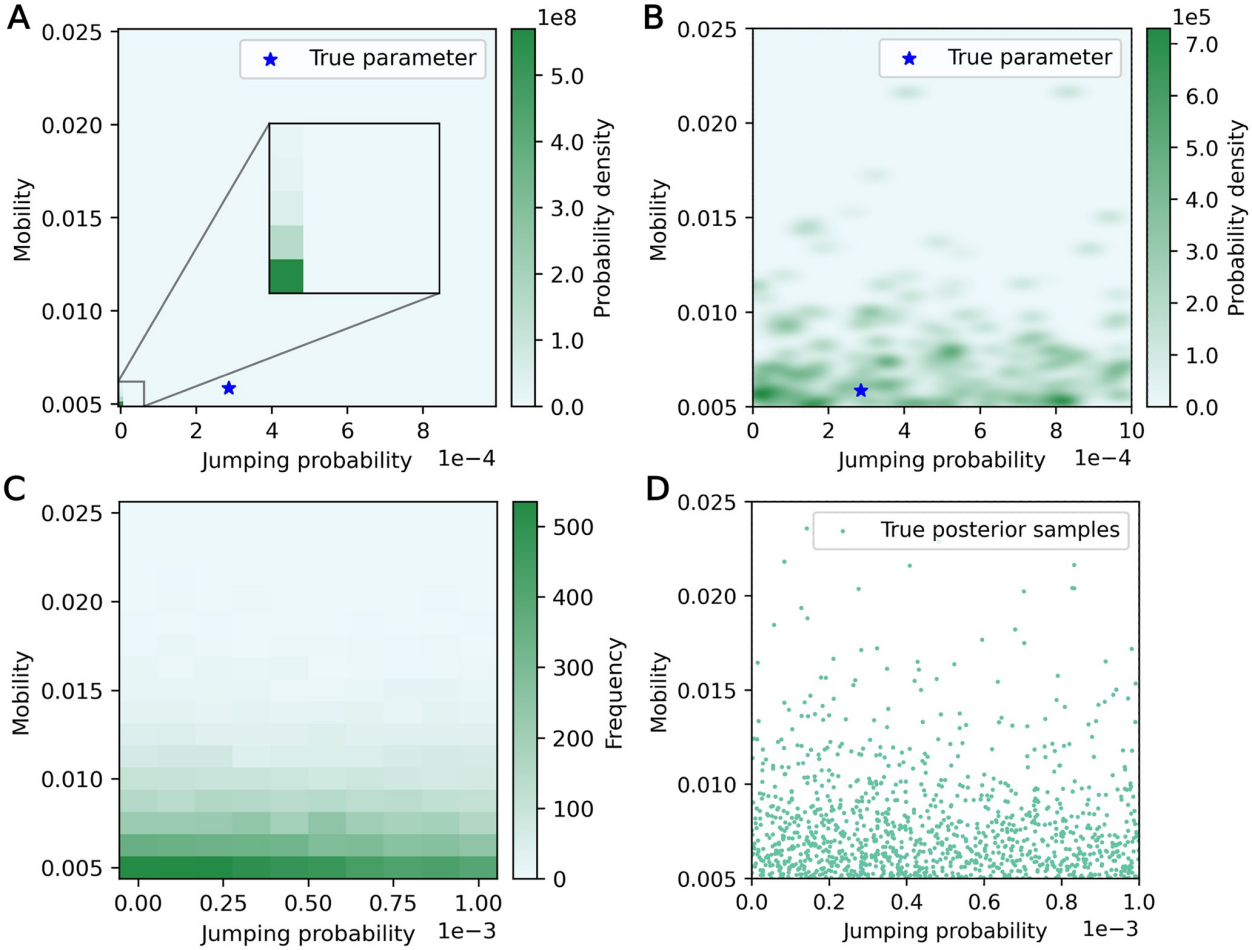
Calibration on a test data sample with zero new infections over the entire simulation. (A) Grid-sampled posterior with calibration method 1. (B) Posterior with calibration method 2. (C) Frequency of matching data (data with zero new infections) across discretely sampled training data set for calibration method 1. (D) Scatter plot of parameter sets resulting in matching data across training data set for calibration method 2.

Beyond aggregate results, we can also identify particular test samples which produced poor posterior inferences when employing calibration method 1. For example, the grid-sampled posterior for a test data sample with zero new infections across all time steps is shown in Fig 16A. The posterior is negligible throughout the domain apart from a very small section in the bottom-left corner, where jumping probability and mobility values are low. The posterior distribution constructed with MCMC is not shown because MCMC failed to converge for this sample, obtaining a small number of unique chain values. Contrast this with the posterior results from calibration method 2 using the ABC rejection algorithm, shown in Fig 16B, where a wider area is assigned high probability density.

We can compare these posteriors to the spread of parameter values that resulted in the same observed data (zero infections across all time steps) within the training data sets for calibration method 1 (Fig 16C) and calibration method 2 (Fig 16D). These distributions represent an approximation of the true posterior (in the case of the training data for calibration method 2, the plotted parameter values are exactly independent and identically distributed samples from the true posterior [39]). Visually, the posterior generated using calibration method 2 reflects the approximate true posteriors more closely than the posterior generated with calibration method 1, despite the approximate true posteriors from both training data sets (Fig 16C and 16D) reflecting a similar spread of parameters. We suggest that this may be due to the assumption of independence between time intervals and sub-populations in the empirical likelihood calculation: by multiplying dependent likelihoods together, what may otherwise be small differences in posterior value could be greatly increased (e.g., if likelihood values were entirely dependent on the first time interval only and remained constant throughout the remaining four time intervals, but were assumed independent, multiplying them together would raise the likelihood to the power of five). This also causes poor MCMC convergence—all of the test data samples with ESS smaller than 2000 in the two-parameter case had similar infection data (≤ 2 total infections across the entire simulation) and similar grid-sampled posteriors (probability density highly concentrated in a small area).

Calibration method 2, which uses a likelihood-free ABC method, results in more favorable SBC histograms than calibration method 1. The data processing for calibration method 2 does not cause any information loss—therefore, the only approximations are in using KDE to construct a posterior PDF, in using a finite number of samples, and in using a non-zero tolerance as defined by *ϵ* and the chosen distance function. As discussed in the Methods section, we expect the latter approximation to yield reasonable results if *ϵ* is sufficiently small. While these approximations appear to have worked well in this scenario, there are some drawbacks to this ABC set-up. For example, because the centroid value *ϵ* sets a fixed proportion of samples with the smallest L2 norms to have non-zero weights regardless of L2 norm magnitude, a test data sample with very few “good” matches will potentially have more biased posterior estimates than other test data samples. Computationally, data generation and distance function evaluations are embarrassingly parallel, and can be computed efficiently. However, this implementation of ABC is ill-suited to high-dimensional problems, since the amount of data and distance function evaluations required will increase very quickly without methods to reduce computational load (note that calibration method 1 would also require large amounts of training data as parameter dimensionality increases).

While calibration method 2 demonstrates better performance than calibration method 1 in our tests, this is not indicative of an inherent superiority of ABC methods or likelihood-free methods in general. The performance results seen here are reflective of suitability for this particular model set-up given the calibration assumptions, implementation, and hyperparameters. In general, choice of calibration method should be tailored to the model and context, and calibration verification can be a step in determining if a method is suitable.

**Fig 17:**
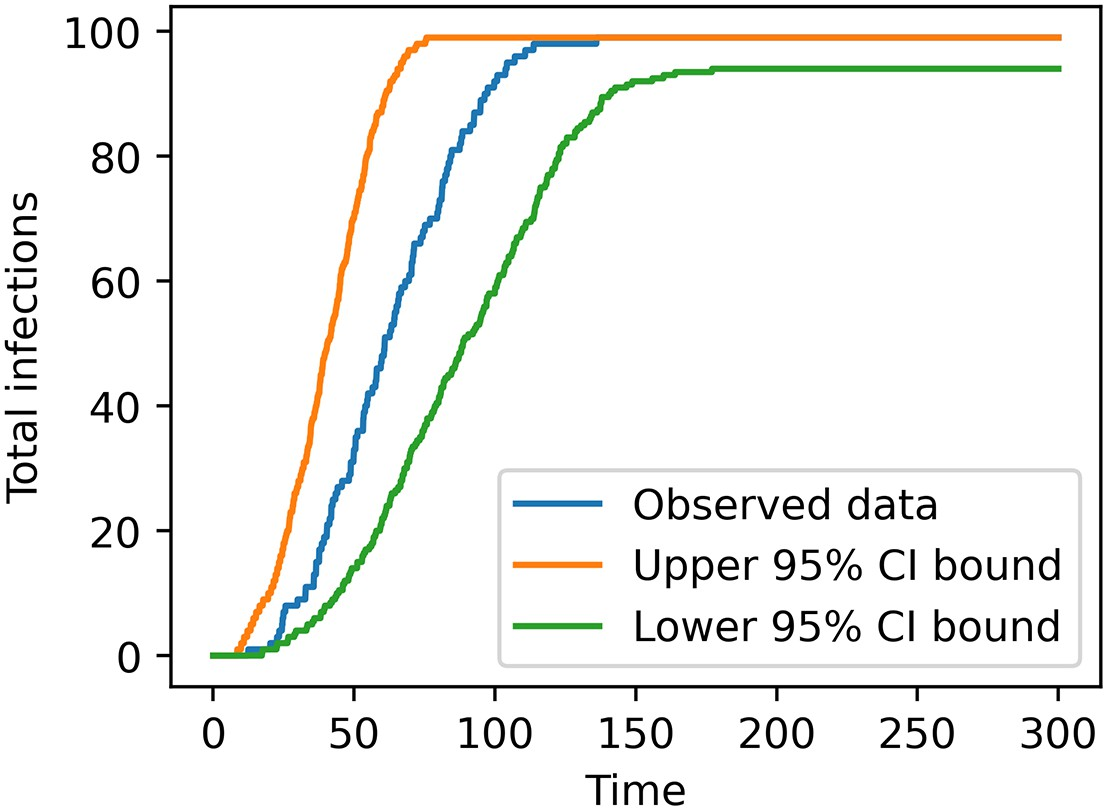
Example of a visual posterior predictive check on synthetic data with calibration method 1.

To illustrate how calibration verification using SBC can help identify hidden calibration issues, we ran a posterior predictive check on synthetic data using calibration method 1 in the one-parameter case, as shown in Fig 17. Some form of posterior predictive check is common in the epidemiology field: researchers estimate a posterior, sample parameter values from the posterior, then generate data with those parameter samples [19, 49]. This allows the predicted data distribution $p(\tilde{x}|x)$ to account for both parameter uncertainty and model stochasticity, which can be compared to the observed data qualitatively or quantitatively. To perform our posterior predictive check, we picked a run from the test data set to serve as synthetic “observed data”, pulled 500 parameter values from the resulting MCMC-generated posterior, then ran the model once with each pulled parameter value (all at different random seeds) to produce posterior predictive data. This data was then summarized into pointwise credible intervals of the accumulated infections over time. We ran this check on 100 synthetic test datasets, and found that the “observed” data fell entirely within the bounds of the 95% interval for 80 of them—In other words, 80% of the checks did not indicate any issue. A tool like SBC, which was able to identify issues with this calibration method, may be valuable in this situation. Additionally, SBC would have added benefits in a real-world data scenario, where data is often limited (there are usually only one or a couple observed datasets available for validation), and model mismatch could make the source of errors unclear.

Additionally, synthetic data tests allow researchers to check if a method is capable of recovering the true values of parameters, which otherwise are often not directly measurable from real-world data (e.g., it would not generally be possible to calculate the ground truth mobility value of a real population in order to compare to the inferred posterior). This is not of significant practical concern for models focused on prediction, but having interpretable parameters is valuable for qualitative understanding of outbreak dynamics [3]. In the cases where researchers wish to use parameter values directly in some way, it may be beneficial to use synthetic data tests to verify that under ideal conditions (perfect model match), the calibration method is able to accurately predict parameter posteriors. While this is not a guarantee of success under real-world data tests, it can help identify preventable issues.

Some limitations of simulation-based calibration include the fact that it cannot prove that a posterior inference procedure is correct, it can only identify incorrect inference procedure with certainty. This is because uniformity of ranks is a necessary but not sufficient condition of a correct posterior inference procedure—if correct, the ranks must be distributed uniformly, but uniform distributions do not prove correctness. Additionally, this method doesn’t test individual test data samples’ posterior predictions, it instead tests on average across many test data samples. It is also limited to analyzing in one parameter dimension at a time.

## Conclusion

We tested two calibration methods on a stochastic agent-based model: calibration method 1 combined an empirical likelihood function with MCMC sampling to perform Bayesian inference, while calibration method 2, used ABC to approximate Bayesian inference with a likelihood-free approach. Both aim to determine the posterior of parameter values given observed data. Performance evaluation was done using simulation-based calibration, a method to determine if posterior inferences on synthetic data are statistically consistent with the parameter values originally used to generate the synthetic data.

Calibration method 1 results in non-uniform SBC rank histograms, which indicates an aggregate issue in the posterior inference procedure. Calibration method 1 is also shown to perform poorly in the specific test case of calibration on a data set with zero new infections across runtime—qualitatively, the predicted posterior appears to be incorrect compared to true posterior samples. In contrast, posteriors from calibration method 2 in this test case are a good approximation of the true posterior. We suggest that the primary cause of poor performance in calibration method 1 are the assumptions and approximations made in the likelihood calculation, including information loss when converting raw data into summary statistics, approximations in empirical PDF construction, and the assumption of independence between time intervals and sub-populations. Importantly, the issues observed here are not inherent to likelihood-based Bayesian inference methods or to MCMC methods in general, but due to specific issues in their implementation. Supporting this, the ESS values for MCMC were sufficiently high for convergence in most cases (the mean ESS values were 10642 for the one-parameter case, 5201 for mobility in the two-parameter case, and 5191 for jumping probability in the two-parameter case) and were visually confirmed to be near convergence by comparison against grid-sampled posterior values, indicating that computational issues were not the primary source of error.

For calibration method 2, a hyperparameter sweep was run in the one-parameter case, with results indicating better performance at smaller KDE bandwidths and centroid values. The two-parameter case was run with the best-performing hyperparameters from the one-parameter case. Overall, this calibration method was not shown to have issues by SBC, as it produced much more uniform SBC rank histograms. While this does not guarantee the correctness of the inference procedure, it serves as a stand-alone check against possible calibration errors. While calibration method 2 appeared to perform well in our tests, it may not be appropriate for high-dimensional calibration problems due to high computational expense. Additionally, the performance of this calibration method is problem- and hyperparameter-dependent, and should be adapted and tested appropriately for use with other models.

Our results indicate that calibration methods for epidemiological models may benefit from stand-alone calibration verification using synthetic data, separate from overall model validation against real-world data. In 100 posterior predictive checks on calibration method 1 using synthetic data, we found that 80% of posterior predictive checks resulted in no clear error. While SBC was able to identify issues with calibration method 1, posterior predictive checks, which are common throughout the field, was not able to identify error in this case. Additionally, validation with real-world data often precludes direct validation of parameter values. Since parameter values may be used for interpretation of outbreak dynamics, it may be valuable to first check for errors in calibration method procedures in a setting where the observed data and model data are both generated by the same process in order to safeguard against underlying issues. A limitation of SBC is that it cannot prove that inference procedures are correct, as the uniformity of ranks is a necessary but not sufficient condition of a correct posterior inference procedure. Additionally, SBC cannot check robustness to choice of prior, assess model correctness, or check the ability of the model to generate data similar to real-world data—however, overall model validation and sensitivity checks can be used in conjunction with SBC to address these points.

## Supporting information

S1 Appendix PDF interpolation. (PDF)

S2 Appendix AMCMC details. (PDF)

S3 Appendix Simulation-based calibration results for calibration method 1 (Bayesian inference using MCMC). (PDF)
