# Supplementary material for: Calibration verification for stochastic agent-based disease spread models
Source: PLoS One. 2024 Dec 10;19(12):e0315429. doi: 10.1371/journal.pone.0315429 (PMC11630602; doi:10.1371/journal.pone.0315429)
Supplement: S1 Appendix — (PDF) [file pone.0315429.s001.pdf]

Calibration verification for stochastic agent-based disease spread models

Maya Horii[1], Aidan Gould[1], Zachary Yun[1], Jaideep Ray[2], Cosmin Safta[2], Tarek Zohdi[1]

1 Mechanical Engineering Department, University of California, Berkeley, Berkeley, California, United States
2 Data Sciences and Computing Department, Sandia National Laboratories, Livermore, CA, United States

* mjhorii@berkeley.edu

S1 Appendix: PDF interpolation

To generate the approximate empirical PDFs, the raw training data (time series of new infections per time step) is first processed to sum the number of infections over each interval, resulting in a set of summary statistics, $\tilde{s}$. The summary statistics consist of values $s_{j,k}$, being the number of new infections in time interval j and sub-population k. We wish to determine the set of PDFs representing the probability of the number of new infections for each time segment and sub-population given a parameter set θ. We will denote the PDFs as $f_{j,k}(s_{j,k}|\theta)$, where j is the represented time interval, and k is the represented sub-population ($j = 1, ..., n$, $k = 1, ..., m$).

For a parameter set θ that is included in the training data, the PDF $f_{j,k}(s_{j,k}|\theta)$ is found by applying Gaussian KDE to the number of new infections in time interval j and sub-population k ($s_{j,k}$) across all recorded stochastic runs. This is repeated for each time interval and sub-population to create the set of PDFs. To facilitate later interpolation, we calculate and store the means ($\tilde{\mu}$) and variances ($\tilde{K}$) of each of these PDFs. However, at some parameter values, there may be no infections across all stochastic runs (for instance, when jumping probability is zero and an infection begins in sub-population 1, any other sub-populations would have zero new infections). This causes an error when trying to generate a KDE, as the covariance matrix of the data is singular. In this case, we replace the KDE with an approximation, as shown in Eq. 1,

$$f_{j,k}^{\sigma=0}(s_{j,k}|\theta) = \begin{cases} \frac{1-(\mu - s_{j,k})/\epsilon}{\epsilon}, & \text{if } \mu - \epsilon \leq s_{j,k} \leq \mu \\ \frac{1-(s_{j,k}-\mu)/\epsilon}{\epsilon}, & \text{if } \mu < s_{j,k} \leq \mu + \epsilon \\ 0, & \text{otherwise} \end{cases} \tag{1}$$

where μ is the mean new infections across the training data set for time interval j and sub-population k (in this case, since data is singular, it is the number of new infections seen in every training data sample for the given j and k), and ϵ is a chosen small value. This represents a PDF with a standard deviation of $\sigma = \sqrt{\epsilon^2/6}$. This approximate PDF allows us to do the interpolation process as usual, preserving the validity of the PDFs and avoiding divide-by-zero errors when calculating transformed coordinates.

One-parameter case interpolation procedure

The following procedure follows Bursal [1]. Say we want to determine the set of PDFs corresponding to parameter set θ that is not contained within the training data set. In the one-parameter case, where θ is a scalar, we can interpolate for the PDFs we want using the training data at the two closest parameter values. Call the lower neighboring parameter $\theta^{(0)}$, and the higher neighboring parameter $\theta^{(1)}$. In general, values relating to the neighboring lower parameter will be denoted with the superscript $^{(0)}$, values relating to the neighboring lower parameter with the superscript $^{(1)}$, and values relating to the final interpolated PDF will have no superscript.

Following [1], first calculate a normalized interpolation coordinate α according to:

$$\alpha = \frac{\theta - \theta^{(0)}}{\theta^{(1)} - \theta^{(0)}} \tag{2}$$

Calculate the approximate mean of the distribution at parameter θ by interpolating between the means of the distributions at neighboring parameters according to:

$$\mu = (1 - \alpha) * \mu^{(0)} + \alpha * \mu^{(1)} \tag{3}$$

Calculate the approximate variance of the distribution at parameter θ by interpolating between the variances of the distributions at neighboring parameters according to Eq. 4. The variance values of $K^{(0)}$ and $K^{(1)}$ are pulled from the set of variances calculated directly from the training data, and therefore may include zeroes.

$$K = (1 - \alpha) * K^{(0)} + \alpha * K^{(1)} \tag{4}$$

We establish a second representation of the variance:

$$\hat{K} = \begin{cases} \epsilon^2/6, & \text{if } K = 0 \\ K, & \text{otherwise} \end{cases} \tag{5}$$

Accordingly, $\hat{\sigma} = \sqrt{\hat{K}}$.

We specify the coordinates at which to sample the interpolated PDF, s, dropping the subscript j, k throughout this section for simplicity. Then, we define the transformed coordinates $s^{(1)}$ and $s^{(0)}$, which are the values at which the distribution corresponding to the upper and lower neighbors will be sampled, respectively. If the coordinates are not transformed, and the values of the interpolated PDF are interpolated naively (such that $f_{j,k}(s|\theta) = (1 - \alpha) f_{j,k}(s|\theta^{(0)}) + \alpha f_{j,k}(s|\theta^{(1)})$), it may cause issues: for example, two normal distributions with different means would interpolate to create a bimodal distribution, instead of a normal distribution with a mean somewhere between the original two.

In calculating the transformed coordinates in Eq. 6, $\hat{\sigma}$ is used in place of σ to avoid divide-by-zero errors when σ is zero. However, this is not of great importance, as the interpolated PDF in that case will be replaced with an approximate, as detailed in Eq. 7 below. Likewise, $\hat{\sigma}^{(0)}$ and $\hat{\sigma}^{(1)}$ are used to avoid multiplying by zero, causing all of the transformed coordinates to sample at a single point repeatedly ($\mu^{(0)}$ or $\mu^{(1)}$, respectively). The result of this is a flat curve, and is obviously not a good interpolation between the two neighboring PDFs.

$$\frac{s - \mu}{\hat{\sigma}} = \frac{s^{(0)} - \mu^{(0)}}{\hat{\sigma}^{(0)}} = \frac{s^{(1)} - \mu^{(1)}}{\hat{\sigma}^{(1)}} \tag{6}$$

Lastly, the interpolated PDF, $f_{j,k}$ can be found using:

$$f_{j,k}(s|\theta) = \begin{cases} f_{j,k}^{\sigma=0}(s|\theta), & \text{if } \sigma = 0 \\ (1-\alpha) * \frac{ds^{(0)}}{ds} f_{j,k}^{\sigma=0}(s^{(0)}) + \alpha * \frac{ds^{(1)}}{ds} f_{j,k}^{(1)}(s^{(1)}), & \text{if } \sigma^{(0)} = 0, \sigma^{(1)} \neq 0 \\ (1-\alpha) * \frac{ds^{(0)}}{ds} f_{j,k}^{(0)}(s^{(0)}) + \alpha * \frac{ds^{(1)}}{ds} f_{j,k}^{\sigma=0}(s^{(1)}), & \text{if } \sigma^{(1)} = 0, \sigma^{(0)} \neq 0 \\ (1-\alpha) * \frac{ds^{(0)}}{ds} f_{j,k}^{(0)}(s^{(0)}) + \alpha * \frac{ds^{(1)}}{ds} f_{j,k}^{(1)}(s^{(1)}), & \text{otherwise} \end{cases} \tag{7}$$

where $f_{j,k}^{\sigma=0}(s|\theta)$ is the approximate stand-in for a PDF with zero-standard deviation defined in Eq. 1, $ds^{(0)}/ds$ is equal to $\sigma^{(0)}/\sigma$, and $ds^{(1)}/ds$ is equal to $\sigma^{(1)}/\sigma$.

It can be verified that the final interpolated PDF is a valid PDF (all values are non-negative and the integral over $s = [-\infty, \infty]$ is unity). It is clear that the values will be non-negative, as they will be the result of linear interpolation between the values of $f_{j,k}^{(0)}(s|\theta)$ and $f_{j,k}^{(1)}(s|\theta)$, which by definition must be non-negative. We can also show that the integral over state space is unity using the definition in Eq. 7 for PDFs with non-zero σ [1]. We use fact (a): $\int_{-\infty}^{\infty} s^{(i)} f^{(i)}(s^{(i)}) ds^{(i)} = \mu^{(i)}$. We drop the subscript on $f_{j,k}$ for simplicity:

$$\int_{-\infty}^{\infty} f(s)ds = (1-\alpha) * \frac{ds^{(0)}}{ds} \int_{-\infty}^{\infty} f^{(0)}(s^{(0)})ds + \alpha * \frac{ds^{(1)}}{ds} \int_{-\infty}^{\infty} f^{(1)}(s^{(1)})ds \tag{8}$$

$$= (1-\alpha) * \int_{-\infty}^{\infty} f^{(0)}(s^{(0)})ds^{(0)} + \alpha * \int_{-\infty}^{\infty} f^{(1)}(s^{(1)})ds^{(1)} \tag{9}$$

$$= 1 - \alpha + \alpha = 1 \tag{10}$$

Additionally, we can verify that the mean is equal to μ. First, we can solve for s from Eq. 6:

$$s = \frac{\hat{\sigma}}{\hat{\sigma}^{(0)}} s^{(0)} + \frac{\mu\hat{\sigma}^{(0)} - \mu^{(0)}\hat{\sigma}}{\hat{\sigma}^{(0)}} \tag{11}$$

$$= \frac{\hat{\sigma}}{\hat{\sigma}^{(1)}} s^{(1)} + \frac{\mu\hat{\sigma}^{(1)} - \mu^{(1)}\hat{\sigma}}{\hat{\sigma}^{(1)}} \tag{12}$$

Then, we verify the mean of the interpolated PDF, using fact (a) and fact (b): $\int_{-\infty}^{\infty} f^{(i)}(s^{(i)}) ds^{(i)} = 1$. In the case that either $\sigma^{(0)}$ or $\sigma^{(1)}$ is 0, the approximate PDF $f_{j,k}^{\sigma=0}$ will be used in place of $f^{(0)}$ or $f^{(1)}$ respectively, consistent with Eq. 7. Facts (a) and (b) are still true under this substitution.

$$\begin{aligned}
\int_{-\infty}^{\infty} sf(s)ds &= (1-\alpha)*\frac{ds^{(0)}}{ds}\int_{-\infty}^{\infty} sf^{(0)}(s^{(0)})ds + \alpha*\frac{ds^{(1)}}{ds}\int_{-\infty}^{\infty} sf^{(1)}(s^{(1)})ds \\
&= (1-\alpha)*\int_{-\infty}^{\infty} sf^{(0)}(s^{(0)})ds^{(0)} + \alpha*\int_{-\infty}^{\infty} sf^{(1)}(s^{(1)})ds^{(1)} \\
&= (1-\alpha)*\Bigg[\frac{\hat{\sigma}}{\hat{\sigma}^{(0)}}\int_{-\infty}^{\infty} s^{(0)}f^{(0)}(s^{(0)})ds^{(0)} \\
&+ \frac{\mu\hat{\sigma}^{(0)}-\mu^{(0)}\hat{\sigma}}{\hat{\sigma}^{(0)}}*\int_{-\infty}^{\infty} f^{(0)}(s^{(0)})ds^{(0)}\Bigg] \\
&+ \alpha*\Bigg[\frac{\hat{\sigma}}{\hat{\sigma}^{(1)}}\int_{-\infty}^{\infty} s^{(1)}f^{(1)}(s^{(1)})ds^{(1)} \\
&+ \frac{\mu\hat{\sigma}^{(1)}-\mu^{(1)}\hat{\sigma}}{\hat{\sigma}^{(1)}}*\int_{-\infty}^{\infty} f^{(1)}(s^{(1)})ds^{(1)}\Bigg] \\
&= (1-\alpha)*(\frac{\hat{\sigma}\mu^{(0)}+\mu\hat{\sigma}^{(0)}-\mu^{(0)}\hat{\sigma}}{\hat{\sigma}^{(0)}}) + \alpha*(\frac{\hat{\sigma}\mu^{(1)}+\mu\hat{\sigma}^{(1)}-\mu^{(1)}\hat{\sigma}}{\hat{\sigma}^{(1)}}) \\
&= \mu
\end{aligned} \tag{13}$$

We can verify the variance of the interpolated PDF similarly. Again, the approximate PDF $f_{j,k}^{\sigma=0}$ will be used in place of $f^{(0)}$ or $f^{(1)}$ if either $\sigma^{(0)}$ or $\sigma^{(1)}$ is 0. Therefore, we can use facts (a), (b), and (c): $\int_{-\infty}^{\infty} {s^{(i)}}^2 f^{(i)}(s^{(i)})ds^{(i)} = {\mu^{(i)}}^2 + \hat{K}^{(i)}$.

$$\int_{-\infty}^{\infty} s^2 f(s)ds = (1-\alpha)*\int_{-\infty}^{\infty} s^2 f^{(0)}(s^{(0)})ds^{(0)} + \alpha*\int_{-\infty}^{\infty} s^2 f^{(1)}(s^{(1)})ds^{(1)} \tag{14}$$

We will examine the first integral term: $\int_{-\infty}^{\infty} s^2 f^{(0)}(s^{(0)})ds^{(0)}$. Recall that $\hat{\sigma}^2 = \hat{K}$.

$$\begin{aligned}
\int_{-\infty}^{\infty} s^2 f^{(0)}(s^{(0)})ds^{(0)} &= (\mu^2 + \frac{{\mu^{(0)}}^2\hat{\sigma}^2}{{\hat{\sigma}^{(0)}}^2} - \frac{2\mu^{(0)}\mu\hat{\sigma}}{\hat{\sigma}^{(0)}})\int_{-\infty}^{\infty} f^{(0)}(s^{(0)})ds^{(0)} \\
&+ (-\frac{2\mu^{(0)}\hat{\sigma}^2}{{\hat{\sigma}^{(0)}}^2} + \frac{2\mu\hat{\sigma}}{\hat{\sigma}^{(0)}})\int_{-\infty}^{\infty} s^{(0)}f^{(0)}(s^{(0)})ds^{(0)} \\
&+ \frac{\hat{\sigma}^2}{{\hat{\sigma}^{(0)}}^2}\int_{-\infty}^{\infty} {s^{(0)}}^2 f^{(0)}(s^{(0)})ds^{(0)} \\
&= \mu^2 + \frac{{\mu^{(0)}}^2\hat{\sigma}^2}{{\hat{\sigma}^{(0)}}^2} - \frac{2\mu^{(0)}\mu\hat{\sigma}}{\hat{\sigma}^{(0)}} - \frac{2{\mu^{(0)}}^2\hat{\sigma}^2}{{\hat{\sigma}^{(0)}}^2} + \frac{2\mu^{(0)}\mu\hat{\sigma}}{\hat{\sigma}^{(0)}} + \frac{{\mu^{(0)}}^2\hat{\sigma}^2}{{\hat{\sigma}^{(0)}}^2} + \frac{\hat{\sigma}^2 S^{(0)}}{{\hat{\sigma}^{(0)}}^2} \\
&= \mu^2 + \frac{\hat{\sigma}^2 S^{(0)}}{{\hat{\sigma}^{(0)}}^2} = \mu^2 + \hat{S}
\end{aligned} \tag{15}$$

It can be similarly shown that $\int_{-\infty}^{\infty} s^2 f^{(1)}(s^{(1)})ds^{(1)} = \mu^2 + \hat{S}$. Therefore,

$$\begin{aligned}
\int_{-\infty}^{\infty} s^2 f(s)ds &= (1-\alpha)*(\mu^2+\hat{S}) + \alpha*(\mu^2+\hat{S}) \\
&= \mu^2 + \hat{S}
\end{aligned} \tag{16}$$

Eqs. 13 and 16 show that the interpolated PDF is valid, and that the mean and variance of the interpolated PDF are consistent with the values calculated in Eq. 3 and Eq. 4 respectively, despite using the approximate PDF $f_{j,k}^{\sigma=0}$ during interpolation for

cases when $\sigma^{(i)} = 0$. However, practically, we can only take a finite number of discrete samples when interpolating, not infinite samples as reflected in the above calculations.

We determine the PDF over the range $s \in [0, N]$, where N is the number of agents in the specified sub-population. Then, we use Eq. 6 to determine the transformed values of coordinates to sample from the high and low PDFs. Then we are able to use Eq. 7 to calculate the values of the interpolated PDF at the chosen grid values. The last step is to re-normalize the PDF over the interval $[0, N]$ to have an integral of 1. Since we use *Gaussian* KDE, the PDF is non-zero over the entire domain, but this is not reflective of what is possible in our simulations, and is corrected through this re-normalization.

The final interpolated PDF is a set of discretely sampled points. For its use in the likelihood function, we linearly interpolate between these samples.

Two-parameter case interpolation procedure

The two-parameter case follows a similar procedure as the one-parameter case. Define: $\theta = [M, J]$, where M is the mobility parameter, and J is the jumping probability parameter. We first identify the neighboring parameters, $M^{(0)}$, $M^{(1)}$, $J^{(0)}$, and $J^{(1)}$. With the set of four corresponding PDFs, we can interpolate along one parameter in state space twice to reduce the set to two PDFs, then once more to get a final PDF.

More concretely, the interpolation procedure can be described:

1. First, we interpolate along the mobility parameter at the lower neighboring jumping parameter (interpolating between $f_{j,k}^{M_{(0)},J_{(0)}}(s|\theta)$ and $f_{j,k}^{M_{(1)},J_{(0)}}(s|\theta)$ to get $f_{j,k}^{J_{(0)}}(s|\theta)$).
2. Then, interpolate along the mobility parameter at the higher neighboring jumping parameter (interpolating between $f_{j,k}^{M_{(0)},J_{(1)}}(s|\theta)$ and $f_{j,k}^{M_{(1)},J_{(1)}}(s|\theta)$ to get $f_{j,k}^{J_{(1)}}(s|\theta)$).
3. Lastly, we can interpolate between $f_{j,k}^{J_{(1)}}(s|\theta)$ and $f_{j,k}^{J_{(0)}}(s|\theta)$ along the jumping parameter to get the final interpolated PDF, $f_{j,k}(s|\theta)$.

Though the interpolated PDF theoretically has an integral of unity over the state space domain when sampled continuously (Eq. 10), we are limited to a finite number of samples, for which this may not hold. Similarly, the K and μ values calculated in steps 1 and 2 are correct over an infinitely fine sampling mesh, but are approximations of the true means and variances of the interpolated PDFs obtained in step 1 and 2 due to the finite, discrete sampling used to construct them. To correct for these two approximations, we first re-normalize the interpolated PDFs obtained in step 1 and 2, then re-evaluate the means and variances of the interpolated PDFs using a trapezoid rule approximation (Eq. 17, Eq. 18). Near $x = 0$, the grid points x must be along a finer mesh than the grid points s along which $f_{j,k}^{J_{(0)}}(s|\theta)$ and $f_{j,k}^{J_{(1)}}(s|\theta)$ are sampled in order to accurately assess the variance. To achieve this with minimal extra computation time, we transform a linear sequence of grid points using a power function with $q = 1.5$ (Eq. 19), and sample along the resulting values. This concentrates mesh density around 0, which is where thin spikes are likely to occur. As shown in Eqs. 17 and 18, we do not reevaluate the mean and variance values of interpolated PDFs with variance K of 0, as these values are already known precisely. We use $\hat{K}$ instead of K to define the new variance value.

$$\mu_{new} = \begin{cases} \mu & \text{if } K = 0 \\ \sum_{k=1}^{N} \frac{[x_{k-1} * f_{j,k}(x_{k-1}|\theta)] + [x_k * f_{j,k}(x_k|\theta)]}{2}(x_k - x_{k-1}) & \text{otherwise} \end{cases} \tag{17}$$

$$K_{new} = \begin{cases} \hat{K} & \text{if } K = 0 \\ \sum_{k=1}^{N} \frac{[(x_{k-1}-\mu_{new})^2 * f_{j,k}(x_{k-1}|\theta)] + [(x_k-\mu_{new})^2) * f_{j,k}(x_k|\theta)]}{2}(x_k - x_{k-1}) & \text{otherwise} \end{cases} \tag{18}$$

$$x_k = \begin{cases} -|x_k|^q, & \text{if } x_k \leq 0 \\ (x_k)^q, & \text{otherwise} \end{cases} \tag{19}$$

In step 3, the mean and variance values of the interpolated PDF will be calculated according to Eq. 3 and Eq. 4, where $\mu^{(0)}$ is equal to μ_{new} of $f_{j,k}^{J_{(0)}}(s|\theta)$ (PDF from step 1), $\mu^{(1)}$ is equal to μ_{new} of $f_{j,k}^{J_{(1)}}(s|\theta)$ (PDF from step 2), $K^{(0)}$ is equal to K_{new} of $f_{j,k}^{J_{(0)}}(s|\theta)$ (PDF from step 1), $K^{(1)}$ is equal to K_{new} of $f_{j,k}^{J_{(1)}}(s|\theta)$ (PDF from step 2). The rest of step 3 proceeds as described in the one-parameter case interpolation section.

Due to the process of transforming the coordinates, the range of points needed for interpolation can be outside the region that is theoretically possible (e.g., if there are only 100 agents in a sub-population, the there is only a non-zero probability for values in the range s = [0,100]). After the first two interpolations are performed (1 & 2 above), we have two PDFs that have been constructed through discrete sampling, and can be evaluated via interpolation. Since we then need to interpolate again between these two PDFs, we need to ensure that the initial discrete samples are taken over a wide enough range to avoid extrapolation, which could lead to negative values, and in turn, invalid PDF values. We do this by sampling along an evenly spaced grid that covers the full range of possible values, along with a margin on both sides. Additionally, we then set the outermost sampling points to be arbitrarily large in magnitude, under the assumption that the points in that region will already be very close to 0 (example of sampling points: s = [-1,000,000, -49, -48, -47...147, 148, 149, 1,000,000]).

References

1. Bursal FH. On interpolating between probability distributions. Applied Mathematics and Computation. 1996;77(2):213–244. doi:https://doi.org/10.1016/S0096-3003(95)00216-2.
