# Supplementary material for: Calibration verification for stochastic agent-based disease spread models
Source: PLoS One. 2024 Dec 10;19(12):e0315429. doi: 10.1371/journal.pone.0315429 (PMC11630602; doi:10.1371/journal.pone.0315429)
Supplement: S2 Appendix — (PDF) [file pone.0315429.s002.pdf]

Calibration verification for stochastic agent-based disease spread models

Maya Horii[1], Aidan Gould[1], Zachary Yun[1], Jaideep Ray[2], Cosmin Safta[2], Tarek Zohdi[1]

1 Mechanical Engineering Department, University of California, Berkeley, Berkeley, California, United States
2 Data Sciences and Computing Department, Sandia National Laboratories, Livermore, CA, United States

* mjhorii@berkeley.edu

S2 Appendix: AMCMC details

The proposal distribution is a Gaussian centered at the current chain value with covariance C_i. The algorithm has two phases: During the non-adaptive period (prior to ν iterations), C_i is adjusted if the fraction of (rejections since last scaled)/(total samples since last scaled) is greater than 0.95 or less than 0.05, in which case the proposal standard deviation is scaled down or up respectively by a chosen scale factor value. During the adaptive period, C_i is updated intermittently (every n_a iterations) using a recursive update shown below in Eqs. 1 and 2,

$$\text{cov}(\theta_0, ..., \theta_i) = \frac{i-1}{i}\text{cov}(\theta_0, ..., \theta_{i-1}) + \frac{i+1}{i^2}(\theta_i - \bar{\theta}_i)(\theta_i - \bar{\theta}_i)^T \tag{1}$$

$$C_i = \begin{cases} s_d\text{cov}(\theta_0, ..., \theta_{i-1}), & \text{if } i \geq \nu \text{ AND cov}(\theta_0, ..., \theta_{i-1}) \text{ is non-singular} \\ s_d\text{cov}(\theta_0, ..., \theta_{i-1}) + s_d\epsilon_c I_d, & \text{if } i \geq \nu \end{cases} \tag{2}$$

where s_d is a parameter, I_d is the identity matrix with dimension d, and ϵ_c is a chosen small value.

We use $s_d = 2.4^2/d$ [1] and $\epsilon_c = 1 \times 10^{-10}$. Initial covariance for the one-parameter case was $C_0 = 0.001$, and for the two-parameter case was $C_0 = 0.001I_d$. The covariance is updated every $n_a = 100$ iterations during the adaptive period.

Algorithm 1 AMCMC Algorithm

```
samples[0] ← init_theta
rej = 0, n = 0,
for k = 0 to n_steps-1 do
    if k = 0 then
        cov ← init_cov
        proposal_cov ← init_cov
        last_update ← 1
    else
        if freq_adapt > 0 & (k + 1)%freq_adapt = 0 & then
            if k < na_period then
                if rej/n > 0.95 then
                    Scale down proposal standard deviation by scale_factor
                else if rej/n < 0.05 then
                    Scale up proposal standard deviation by scale_factor
                end if
                rej = 0, n = 0
            else
                Recompute covariance matrix recursively with data from
                samples[last_update:last_update+freq_adapt]
                last_update ← last_update+freq_adapt
                if cov is singular then
                    cov += (identity matrix)*cov_eps
                end if
                Scale proposal covariance by s_d
            end if
        end if
    end if
    Generate proposal theta.
    if theta is within bounds then
        Calculate likelihood, accept or reject based on Metropolis procedure.
    else
        Reject proposal.
    end if
    if Proposed theta is accepted then
        samples[k+1]=theta
    else
        samples[k+1]=samples[k]
        rej += 1
    end if
    n += 1
end for
```

References

1. Haario H, Saksman E, Tamminen J. An Adaptive Metropolis Algorithm. Bernoulli. 2001;7(2):223. doi:10.2307/3318737.
