# Supplementary material for: Calibration verification for stochastic agent-based disease spread models
Source: PLoS One. 2024 Dec 10;19(12):e0315429. doi: 10.1371/journal.pone.0315429 (PMC11630602; doi:10.1371/journal.pone.0315429)
Supplement: S3 Appendix — (PDF) [file pone.0315429.s003.pdf]

Calibration verification for stochastic agent-based disease spread models

Maya Horii[1], Aidan Gould[1], Zachary Yun[1], Jaideep Ray[2], Cosmin Safta[2], Tarek Zohdi[1]

1 Mechanical Engineering Department, University of California, Berkeley, Berkeley, California, United States
2 Data Sciences and Computing Department, Sandia National Laboratories, Livermore, CA, United States

* mjhorii@berkeley.edu

S3 Appendix: Simulation-based calibration results for calibration method 1 (Bayesian inference using MCMC)

One-parameter case

After collecting 75,000 MCMC iterations, discarding 25,000 for burn-in, then thinning based on the ESS of the chain, the shortest thinned chain contains 60 iterations. By setting $L = 50$, we do not need to use the rank approximation (Eq. 6) on any samples. Therefore, the results in Fig 11A directly follow the thinning and truncation procedure presented in Talts et al. [1]. A χ^2 test performed over the $L + 1$ bins returns a p-value of $1.6 * 10^{-191}$, which rejects the null hypothesis of uniformity.

Two-parameter case

In the two-parameter case, the shortest thinned chains contain 8 iterations. However, for many samples (93 out of the total 1666), it was not possible to find an ESS value that eliminated auto-correlation — even if we use $L = 8$ to eliminate the need for a rank approximation, we expect that auto-correlation would still be present. We present the results with $L = 8$ in Fig 1.

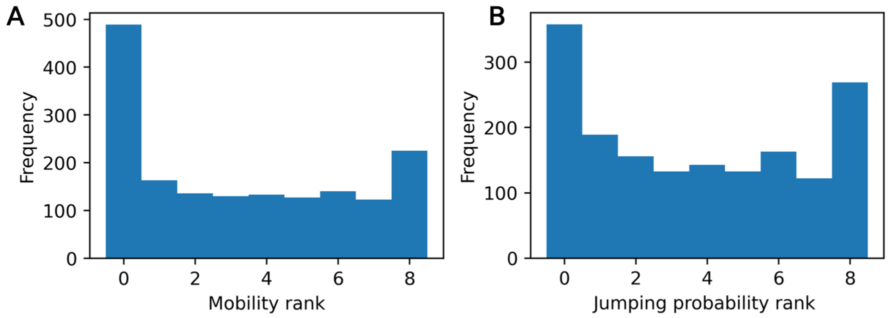

Fig 1. SBC results for calibration method 1. (A) SBC histogram in the two-parameter case over the mobility parameter. (B) SBC histogram in the two-parameter case over the jumping probability parameter.

The SBC histograms for both the mobility and jumping probability (Fig 1) display non-uniformity (from a χ^2 test, mobility p-value $= 2.78 * 10^{-125}$, jumping probability p-value $= 3.81 * 10^{-53}$), but it may not be due solely to incorrect posterior inference, since there is still some auto-correlation in the posterior samples $\theta_1, ...\theta_L$. However, given the level of non-uniformity, the small number of auto-correlated samples, and the similar failure in the one-parameter case, it appears likely that the non-uniformity observed in the SBC histogram is not solely due to the remaining auto-correlation in samples, but also in part due to incorrect posterior inference.

References

1. Talts S, Betancourt M, Simpson D, Vehtari A, Gelman A. Validating Bayesian Inference Algorithms with Simulation-Based Calibration; 2020. Available from: `http://arxiv.org/abs/1804.06788`.
